# Data‐Driven Printability Modeling of Hydrogels for Precise Direct Ink Writing Based on Rheological Properties

**DOI:** 10.1002/advs.202507639

**Published:** 2025-07-03

**Authors:** Eun Hui Jeong, Jiho Choi, Han Bi Park, Ji Woo Lee, Seo Yeon Bae, Byoung Soo Kim, ChangKyu Yoon, Jun Dong Park

**Affiliations:** ^1^ Department of Chemical and Biological Engineering Sookmyung Women's university Seoul 04310 Republic of Korea; ^2^ Department of Mechanical Systems Engineering Sookmyung Women's university Seoul 04310 Republic of Korea; ^3^ Bio‐convergence R&D Division Korea Institute of Ceramic Engineering and Technology (KICET) Chungbuk 28160 Republic of Korea; ^4^ Institute of Advanced Materials and Systems Sookmyung Women's University Seoul 04310 Republic of Korea

**Keywords:** 3D printability, 3D/4D printing, machine learning, soft robotics, stimuli‐responsive

## Abstract

Hydrogels are gaining significant attention in soft robotics and electronics due to their favorable mechanical properties and sustainability. While hydrogel inks enable three‐dimensional (3D) printing as a key fabrication technique, the relationship between their rheological behavior and printability remains insufficiently understood. This study quantitatively examines this correlation through a rheology‐printability database of 150 3D‐printed hydrogels analyzed via machine learning. The database includes nonlinear rheological metrics, such as large‐amplitude oscillatory shearing (LAOS), which mimic real 3D printing conditions involving repeated flow and stoppage. Printability is quantitatively evaluated in horizontal and vertical directions and inconsistency through image analysis of 3D printed structures. A predictive model for printability is developed using Random Forest regression, achieving reliable predictions within a 10% margin. Permutation importance analysis suggested that horizontal printability is primarily influenced by variables related to post‐extrusion recovery and relaxation process, whereas vertical printability is mainly governed by viscous responses under high‐strain‐rate flow through the nozzle. Overall, this study provides quantitative insights into the intricate relationship between hydrogel rheology and 3D printability, paving the way for the sustainable design of hydrogel inks and their 3D printing processes for the precise fabrication of soft robotics structures and electronics.

## Graphical abstract

A predictive model for 3D printability is developed by integrating rheological analysis, including the Large Amplitude Oscillatory Shear (LAOS) test, with machine learning. With prediction errors under 10%, the model shows that post‐extrusion recovery controls horizontal printability, while high‐strain‐rate nozzle flow dictates vertical printability. These insights clarify rheology–printability relationships in 3D printing for soft robotics and electronics.

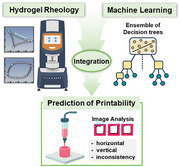

## 1 Introduction

The transition toward sustainable materials has gained significant momentum across various fields, driven by growing environmental concerns and the demand for greener alternatives. Among these materials, hydrogels have emerged and evolved as sustainable materials, characterized by key features such as recyclability, reusability, customizability, and environmental compatibility.^[^ ^1^, ^2^, ^3^, ^4^ ^]^ Their favorable mechanical characteristics, combined with recent advances in three‐dimensional (3D) printed hydrogel systems, further underscore their potential, particularly in emerging fields such as soft robotics and flexible electronics.^[^ ^5^, ^6^, ^7^, ^8^, ^9^, ^10^, ^11^, ^12^, ^13^, ^14^, ^15^ ^]^ Concurrently, the advent of additive manufacturing techniques such as 3D printing has provided a powerful platform for the precise fabrication of hydrogel‐based systems. 3D printing offers significant advantages, including enhanced materials utilization, reduced processing time, and the ability to fabricate application‐specific architectures.^[^ ^5^, ^16^, ^17^, ^18^ ^]^ Moreover, 3D printing enables high‐resolution control over spatial material distribution and complex geometrical design, thereby expanding the functional versatility of hydrogel constructs.^[^ ^18^, ^19^, ^20^, ^21^ ^]^ Collectively, these attributes position 3D printing as a pivotal strategy for the advanced processing of hydrogels in next‐generation soft robotics and electronics.

During the 3D printing process, hydrogel inks experience a range of complex flow environments, including slow deformation and flow in the reservoir, rapid, high‐shear deformation as they pass through the nozzle, and static retention after deposition.^[^ ^22^, ^23^, ^24^, ^25^ ^]^ Under these complex flow conditions, hydrogel inks, which consist of polymers and various additives, undergo microstructural changes, such as network disruption and chain alignment, leading to intricate rheological behaviors.^[^ ^26^, ^27^, ^28^ ^]^ The interaction of these rheological behaviors with the geometric and temporal constraints of the 3D printing process results in complex flow phenomena that critically impact print fidelity, resolution, and overall efficiency. As such, a comprehensive understanding of hydrogel ink rheology under processing‐relevant conditions is critical for optimizing materials performance and enabling precision fabrication in advanced applications.

Despite the critical role of rheological analysis in hydrogel‐based additive manufacturing, systematic investigations in the relationship between rheological properties and 3D printability remain limited. Although many previous studies on the 3D printing of hydrogel have characterized the rheological properties of hydrogel inks, they primarily rely on conventional rheological metrics, such as viscosity, viscoelastic modulus (*G*′ and *G*′′), and yield stress(σ_*y*_).^[^ ^29^, ^30^, ^31^, ^32^, ^33^, ^34^, ^35^, ^36^, ^37^, ^38^ ^]^ Some efforts have employed more advanced rheological tests, such as three‐interval thixotropy tests (3ITT).^[^ ^39^, ^40^, ^41^, ^42^ ^]^ However, these metrics still fall short in capturing the full spectrum of highly nonlinear and complex rheological transitions that hydrogels experience during realistic 3D printing. The lack of a quantitative framework for describing printing accuracy is another factor hindering the understanding of the rheology‐printability correlation. While most studies have assessed printability qualitatively, primarily through visual observation of printed structures,^[^ ^43^, ^44^, ^45^ ^]^ a deeper understanding of the rheology‐printability correlation necessitates the quantification of printability.

A final and critical challenge in uncovering the correlation between rheology and printability lies in accurately modeling their inherently complex, multivariate, nonlinear relationship. Printability can be regarded as a multivariable function of rheological metrics, where the relationship is inherently nonlinear and cannot be adequately captured by simple linear correlations. Although prior studies often demonstrate qualitative or simplified linear correlations, such as positive or negative trends, these findings remain ambiguous and insufficient without a profound quantitative understanding.^[^ ^11^, ^46^, ^47^ ^]^ Recently, machine learning techniques have emerged as a powerful alternative for analyzing and interpreting complex datasets. As a subset of Artificial Intelligence, machine learning automates the efficient and reproducible analysis of large and complex datasets. Moreover, machine learning is extremely useful in statistically learning from data, even in scenarios where data is limited. It has become an essential tool for identifying complex patterns between variables and building predictive models that surpass human analytical capabilities. This widespread adoption of machine learning for handling datasets extends across many research fields,^[^ ^48^, ^49^, ^50^, ^51^, ^52^, ^53^ ^]^ including economics, biological sciences, and medicine, demonstrating how it accelerates scientific discovery and technological innovation across various industries.

**Figure 1:**
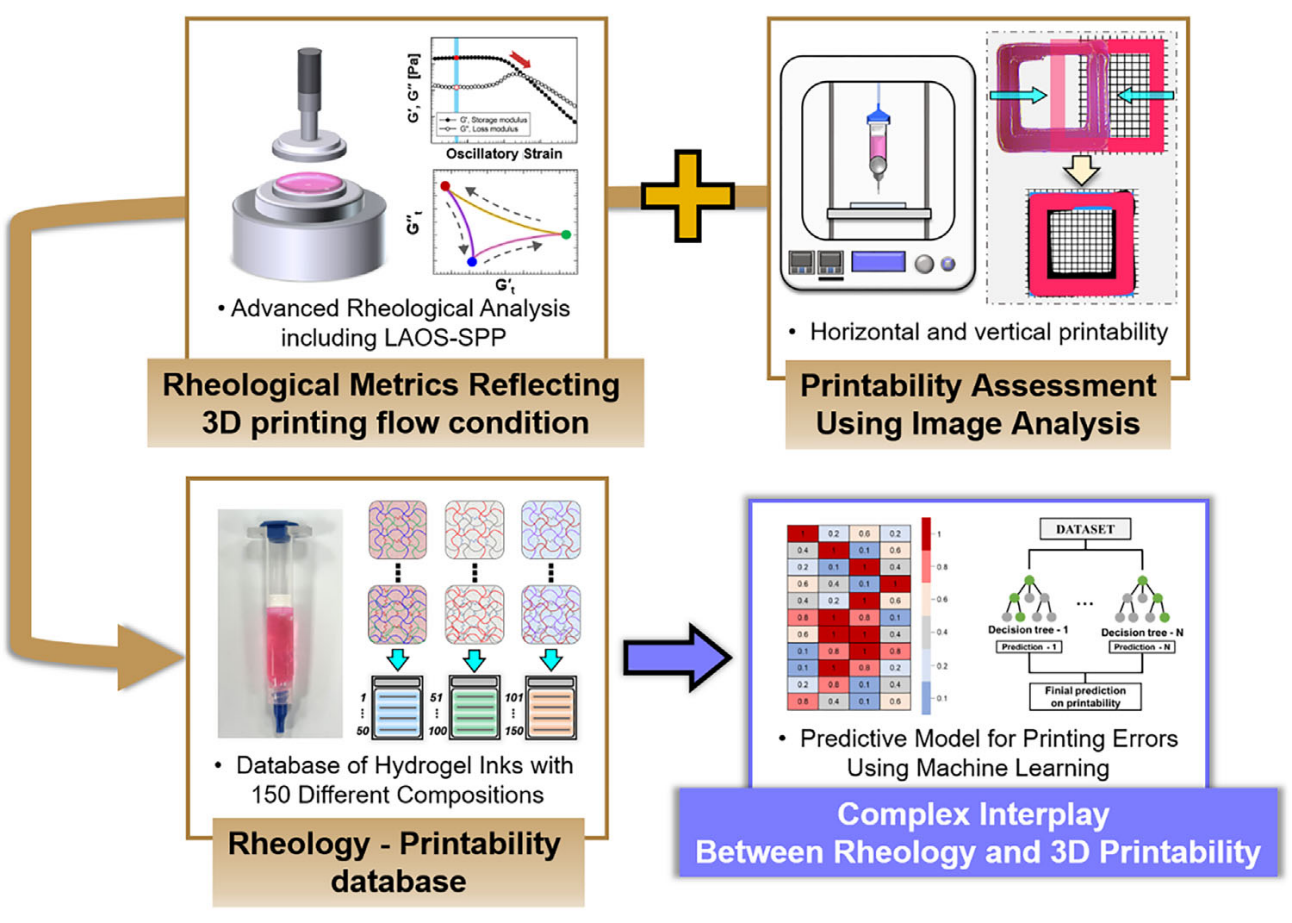
Strategic approaches for investigating the correlation between the rheology of hydrogel and 3D printability.

This study systematically investigates the complex relationship between the rheological properties of hydrogel inks and their 3D printability, addressing key limitations in current research, as outlined in **Figure** **1**. To capture the nonlinear, multivariate nature of this relationship, we developed a machine learning model based on Random Forest regression, trained on a comprehensive database comprising 150 hydrogels with diverse compositional profiles. We employed a suite of rheological metrics obtained under conditions that closely simulate the dynamic flow environment of 3D printing. Notably, the rheology database incorporates nonlinear rheological measurements derived from large‐amplitude oscillatory shearing (LAOS) and the sequence of physical processes (SPP) analysis,^[^ ^54^, ^55^, ^56^, ^57^ ^]^ which more faithfully reflects the cyclic flow‐rest dynamics inherent to the 3D printing process by capturing both viscous and elastic responses thereby addressing the limitations of thixotropy‐based metrics that primarily focus on viscous behavior. The LAOS‐SPP analysis has proven effective in capturing complex rheological properties across a range of materials, including slurries, suspensions, and cosmetics.^[^ ^58^, ^59^, ^60^, ^61^ ^]^ Under LAOS, materials undergo a wide range of rheological transitions, including flow, yielding, and relaxation,^[^ ^62^, ^63^ ^]^ which mirror the rheological changes hydrogels experience during 3D printing. SPP analysis of the stress response during LAOS provides temporally resolved insights into rheological behavior, expressed in terms of transient elastic and viscous moduli. These transient moduli effectively characterize rheological behaviors during 3D printing and correlate strongly with hydrogel printability, based on the analogous flow conditions between LAOS and the 3D printing process. Another key innovation of this study is the implementation of a quantitative printability metric based on high‐resolution image analysis. While previous studies often relied on qualitative assessments of printed shapes by merely observing printed shapes and categorizing outcomes as success or failure,^[^ ^64^, ^65^, ^66^ ^]^ this study quantifies structural printability in both horizontal and vertical dimensions, ensuring a precise and objective representation of printability.

Our predictive model, built upon a comprehensive rheological database, exhibited reliable performance in forecasting hydrogel printing outcomes with high accuracy. Specifically, the model was able to predict both horizontal and vertical print fidelity within a 10% margin of error, relying solely on rheological measurements of hydrogel formulations. Beyond predictive capability, we employed permutation importance analysis to elucidate the relative influence of individual rheological metrics on 3D printing performance. To contextualize these findings, we interpreted these metrics in the context of flow conditions across four key printing stages: 1) reservoir storage, 2) initial flow through the contraction channel, 3) high‐strain‐rate deformation during nozzle passage, and 4) post‐extrusion recovery and relaxation. This analysis not only uncovers the complex relationships between hydrogel rheology and printability but also demonstrates how data‐driven approaches, incorporating feature selection informed by advanced rheological knowledge, can elucidate the interplay between rheological properties and fluid processes.

## 2 Results and Discussion

### 2.1 Simplification and Segmentation of the 3D Printing Process

**Figure 2:**
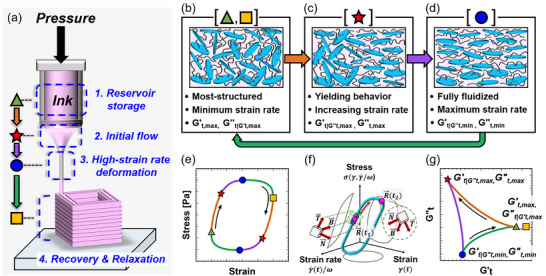
Comparison of rheological transitions during LAOS and the four subprocesses in the 3D printing process. a) Subdivision of the 3D printing process into four distinct subprocesses. b)‐d) Illustration of microstructural changes and rheological transitions in a hydrogel ink composed of Laponite and N‐isopropyl acrylamide (NIPAM) at four marked points during LAOS and corresponding subprocesses in the 3D printing process. e) Elastic Lissajous curve under LAOS. f) Representation of rheological states under LAOS as a trajectory in a 3D space of strain (***γ***), dimensionless strain rate ($\dot{\gamma }/\omega$), and stress (***σ***). $\vec{R}\left(t_{1}\right)$ and $\vec{R}\left(t_{2}\right)$ indicates the rheological states at the two different times ***t***_1_ and ***t***_2_. g) Cole‐Cole plot from the SPP analysis.

The rheological behavior of hydrogel inks is typically controlled through the incorporation of rheological additives that induce distinct microstructures, thereby tailoring the material's flow properties for optimal performance in 3D printing. In this study, hydrogel inks were categorized into three distinct types based on the type of rheological additives employed, as detailed in the experimental section. These inks were selected because they are commonly used in extrusion‐based 3D printing and satisfy the key process requirements, including shear‐thinning behavior for smooth extrusion, excellent biocompatibility for biological and soft robotics applications, tunable viscoelastic properties through compositional adjustments, and stable processability during printing.^[^ ^18^, ^67^, ^68^ ^]^ Specifically, two of the hydrogel ink formulations incorporate Laponite nanoclay, while the third uses Carbomer as a rheological additive. Laponite nanoclay features positively charged edges and negatively charged planes.^[^ ^69^, ^70^ ^]^ Upon dispersion in water, it forms a “house‐of‐cards” structure.^[^ ^71^, ^72^ ^]^ The planes of this structure containing oxygen atoms form hydrogen bonds with the amide groups of NIPAM or the amine groups of acrylamide, creating the network structures.^[^ ^73^, ^74^, ^75^ ^]^ In contrast, Carbomer consists of crosslinked polyacrylic acid (PAA) chains, contains carboxylate anions, and exists as microgel particles when dispersed and ionized in water.^[^ ^76^, ^77^ ^]^ These microgels swell and form a closely packed structure. The characteristic microstructures of these additives impart the necessary rheological properties, such as yield stress, required for effective 3D printing. In the 3D printing process, the hydrogel inks are intermittently dispensed to achieve the desired feature, subjecting them to repeated flow and stoppage conditions. During dispensing, hydrogel inks undergo a printing process that can be divided into four subprocesses as illustrated in **Figure** **2**a: 1) reservoir storage, 2) initial flow through the contraction channel, 3) high‐strain‐rate deformation during nozzle passage, and 4) post‐extrusion recovery and relaxation. At each stage, the characteristic microstructures within hydrogel inks evolve in response to the prevailing flow conditions, leading to distinct corresponding rheological behaviors that critically influence the fidelity and resolution of the 3D printed construct.

During the reservoir storage phase (denoted by the green triangle in Figure 2a), hydrogel inks remain most structured and relaxed state with the highest elasticity due to the large diameter geometry, which minimizes strain rate. For example, the network structure formed by Laponite remains undisturbed during this process, as illustrated in Figure 2b. As the hydrogel inks begin to flow through the contracting channel below the reservoir (indicated by the red star in Figure 2a), where strain and strain rate increase, the characteristic microstructure within the hydrogel inks begins to deform and undergo destruction, accompanied by rheological yielding behavior. This process can be related to the breakage and shear‐induced alignment of Laponite, as shown in Figure 2c. Upon passing through the narrow printing nozzle (marked by the blue circle in Figure 2a), the hydrogel ink becomes fully fluidized due to the high‐strain‐rate and is then extruded. This corresponds to Figure 2d, where most of the Laponite network structure in the hydrogel ink is broken and aligned in the flow direction. Once outside the nozzle and deposited onto the substrate (represented by the yellow square in Figure 2a), the hydrogel inks are exposed to a free surface without shear forces, allowing them to relax and recover their microstructure, thereby retaining their printed form. For hydrogel inks containing Laponite, this subprocess corresponds to the transition from Figure 2b–d, during which the Laponite network structure and rheological properties are restored.

### 2.2 Analogy Between Large Amplitude Oscillatory Shear (LAOS) and the 3D Printing Process

From the perspective of flow conditions and rheological transitions, the 3D printing process is analogous to LAOS. In LAOS, strain (γ) and strain rate ($\dot{\gamma }$) follow sinusoidal functions, γ_0_sin (ω*t*) and γ_0_cos (ω*t*), respectively, with a large strain amplitude (γ_0_).^[^ ^55^, ^57^, ^63^ ^]^ This process subjects hydrogel inks to repeated cycles of strong flow and relaxation, mirroring the dispensing and stoppage phases that occur during 3D printing. Specifically, the rheological transitions observed in hydrogel inks under LAOS can be mapped onto the subprocesses of 3D printing, providing deeper insights into their rheological behavior. In Figure 2a,e, the corresponding subprocesses of 3D printing and LAOS are highlighted identically, illustrating their direct analogy. Figure 2e displays the elastic Lissajous curve, illustrating stress as a function of strain under LAOS. At the green triangle point in Figure 2e, the strain rate is nearly zero, allowing hydrogel inks to relax from accumulated strain and recover their network structure. Here, it should be noted that although the imposed strain at the green triangle point is nearly at its maximum, the accumulated strain in the hydrogel ink is minimal.^[^ ^57^, ^58^ ^]^ This state corresponds to the reservoir storage stage in the 3D printing, where the hydrogel is in its most relaxed state during repeated dispensing and stoppage due to the minimal strain rate. As flow reverses and the strain rate increases in the opposite direction (from the green triangle to the red star in Figure 2e), hydrogel inks exhibit elastic yielding behavior, where the hydrogel ink's network structure begins to rupture. This elastic yielding behavior corresponds to the second subprocess, in which the hydrogel inks initially flow through a contraction zone, experiencing increasing accumulated strain and strain rate due to the contracting geometry. Between the red star and the blue circle in Figure 2e, the strain rate increases to its maximum, subjecting the hydrogel inks to the strongest flow conditions. This leads to fully fluidization, where the hydrogel inks transition to viscoplastic flow in the vicinity of the blue circle. This transition corresponds to the third subprocess, characterized by high‐strain‐rate deformation during nozzle passage in the 3D printing. As the strain approaches another maximum in the opposite direction, marked by the yellow square in Figure 2e, the strain rate begins to decrease. In this phase, hydrogel inks recover their network structure, eventually returning to the same state as the green triangle. This process resembles the fourth subprocess of 3D printing, previously described as the post‐extrusion recovery and relaxation. These rheological transitions occur within a half oscillation cycle and are repeated symmetrically during the second half of the cycle.

### 2.3 Rheological Features for Predictive Modeling of Hydrogel Ink 3D Printability

The SPP analysis of stress responses under LAOS provides temporally resolved information regarding the rheological transitions occurring during oscillation.^[^ ^54^, ^55^, ^56^, ^57^, ^58^, ^59^, ^60^, ^61^ ^]^ Given the analogy between LAOS flow conditions and those experienced during 3D printing, the LAOS‐SPP metrics can be effectively utilized to indirectly characterize the rheological transitions of hydrogel inks throughout the 3D printing process. Viscoelastic materials, including hydrogels, exhibit a complex stress response that depends on both strain and strain rate. Thus, SPP analysis considers the stress response under the oscillatory shearing as a function of strain and dimensionless strain rate, $\sigma (\gamma \left(t\right),\;\dot{\gamma }\left(t\right)/\omega )$. Here, it should be noted that dimensionless strain rate is employed rather than strain rate for compatibility with conventional viscous modulus (*G*′′). Accordingly, the stress response (σ) to LAOS forms a trajectory in strain (γ)‐strain rate ($\dot{\gamma }/\omega$)‐stress space as shown in Figure 2f. By applying the Frenet‐Serret framework, which incorporates tangent ($\vec{T}$), normal ($\vec{N}$), and binormal ($\vec{B}$) vectors, an osculating plane can be defined at an arbitrary point along the trajectory (sky‐blue solid line). Based on the osculating plane, the partial derivatives of stress with respect to strain ($\frac{\partial \sigma }{\partial \gamma }$) and strain rate ($\frac{\partial \sigma }{\partial \dot{\gamma }\left(t\right)/\omega }$) are obtained. These derivatives correspond to the transient elastic modulus $G_{t}^{\prime }\left(t\right)$ and transient viscous modulus 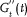, respectively. The complete derivation is provided in the Experimental section and referenced elsewhere.^[^ ^55^, ^57^ ^]^

The transient moduli can be regarded as time‐dependent analogues of the conventionally used dynamic moduli (*G*′ and *G*′′).^[^ ^57^, ^59^ ^]^ Since $G_{t}^{\prime }\left(t\right)$ and 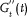 indicate the partial derivatives of the stress with respect to strain and dimensionless strain rate, respectively, they capture the transient influence of strain and strain rate on the stress response separately, aligning with the definitions of differential elastic and viscous moduli. The rheological transition during LAOS is typically represented through changes in the transient moduli values using a Cole‐Cole plot, as demonstrated in Figure 2g. This plot can be interpreted as the trajectory of a point, where the horizontal and vertical coordinates correspond to $G_{t}^{\prime }\left(t\right)$ and 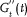, respectively. Typically, the Cole‐Cole plot of most materials, including all the hydrogels studied in this work, exhibits a deltoid shape under LAOS due to the dominance of the third harmonic in the Fourier spectrum of the stress response,^[^ ^57^ ^]^ unless the material exhibits distinctive rheological behavior such as two‐step yielding.^[^ ^58^ ^]^ By corresponding the movements of points on the elastic Lissajous curve shown in Figure 2e and the Cole‐Cole plot given in Figure 2g, the time‐dependent rheological state of the material during the oscillation can be identified, with the corresponding regions highlighted using the same colors and markers.

We selected the transient moduli at the three vertices of the deltoid curve, as marked in Figure 2g, as metrics to characterize the rheological behavior of each hydrogel ink in the 3D printing process, and utilized them as feature variables in the machine learning model to predict printability. Specifically, the transient moduli at the green triangle point ($G_{t,max}^{\prime }$, 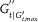) in Figure 2g—where strain rate is nearly zero and the transient elastic modulus is the maximum in the Cole‐Cole plot—represent the most structured rheological state during LAOS, corresponding to the reservoir storage state under repeated flow and stoppage conditions in the 3D printing. The trajectory from the green triangle point to the red star point (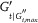, 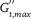) in Figure 2g indicates an initial transition from elastic to viscous behavior, characterized by a decrease in $G_{t}^{\prime }\left(t\right)$ and an increase in 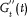. This transition corresponds to the subprocess involving the initial flow through the contraction channel. In the subsequent transition from the red star point to the blue circle point (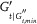, 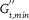), where the strain rate reaches its maximum, a viscous yielding behavior emerges, indicated by a decrease in 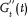. This behavior is associated with the third subprocess, specifically the viscoplastic flow through the nozzle. Lastly, the path from the blue circle to the yellow square in Figure 2g, during which the strain rate again decreases toward zero, shows the recovery of both $G_{t}^{\prime }\left(t\right)$ and 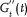 to values equivalent to those at the green triangle point. This corresponds to the fourth subprocess of the post‐extrusion recovery and relaxation, once free from shearing forces.

**Figure 3:**
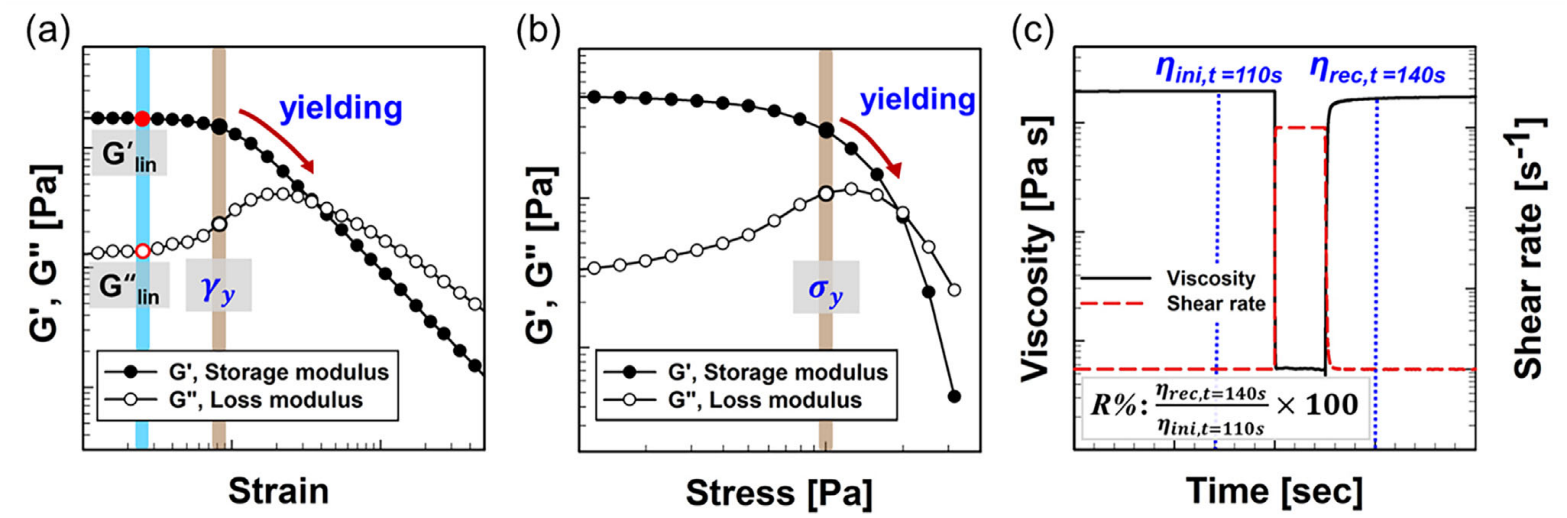
Feature variables from conventional rheological measurements. a) Linear elastic and viscous moduli (*G*′_*lin*_ and *G*′′_*lin*_) and yield strain (γ_*y*_) obtained from the dynamic stain amplitude sweep. b) Yield (σ_*y*_) stress measured from the dynamic stress amplitude sweep. c) Recovery rate of viscosity (*R*%) measured from the three‐interval thixotropy test (3ITT).

Previous research has highlighted that hydrogel inks suitable for direct ink writing (DIW) typically require sufficient yield stress, shear‐thinning behavior, and an appropriate viscoelastic balance to enable smooth extrusion and maintain structural integrity after deposition.^[^ ^78^, ^79^, ^80^, ^81^ ^]^ This prior knowledge provided a useful basis for designing the experimental system and selecting rheological metrics relevant to 3D printability in this study, while also serving as a rough filtering step to exclude obviously unsuitable systems in the early stage. Accordingly, rheological metrics obtained from three conventional measurements were included as feature variables: linear elastic modulus $\left(G_{lin}^{\prime }\right)$, linear viscous modulus 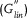, and the yield strain (γ_*y*_) obtained from the strain amplitude sweep in **Figure** **3**a; the yield stress (σ_*y*_) from the stress amplitude sweep in Figure 3b; and recovery rate of viscosity from 3ITT in Figure 3c, denoted as *R*%. The linear elastic and viscous moduli characterize the rheological properties of hydrogel inks in an undisturbed quiescent state, which may be relevant to the reservoir storage process or the post‐extrusion recovery and relaxation process. The yield strain and stress are defined as the strain or stress amplitude at which hydrogel inks begin to exhibit more than 5% of decrease in elastic modulus compared to their values in the linear regime. These metrics indicate the maximum strain and stress that hydrogel inks can endure before yielding and are associated with the post‐extrusion recovery and relaxation process. The recovery rate of viscosity is defined as the ratio of viscosity at the initial strain rate of 0.1s^−1^ (before exposure to a high‐strain‐rate of 100*s* ^−1^) to the viscosity at 0.1s^−1^ after 10 s of high‐strain‐rate application. Such flow conditions were determined based on the effective strain rate in the nozzle, with calculation details provided in the Experimental Section. This recovery rate reflects the ability of the hydrogel inks to regain their rheological properties in response to flow condition changes, such as high‐strain‐rate deformation during the nozzle passage process to post‐extrusion on the substrate. Further details regarding the rheological measurements can be found in the Experimental section.

**Table** **1** summarizes the rheological metrics employed in this study and their interpretation in the context of 3D printing subprocesses. The specific roles of the 11 rheological metrics are described in detail. Each metric reflects distinct responses of hydrogel inks such as flow initiation, fluidization, and structural recovery. Starting with conventional rheological metrics, high values of $G_{lin}^{\prime }$ and 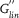
​ indicate that the ink can stably maintain its structure either in a quiescent state or after extrusion with minimal deformation. A large yield strain (γ_*y*_​) and yield stress (σ_*y*_​) suggest that the ink possesses strong structural integrity, capable of resisting uncontrolled flow caused by gravity or vibration and supporting the printed shape against collapse. The recovery rate (R%) obtained from the 3ITT test quantifies how quickly the ink regains its initial viscosity after experiencing high‐strain‐rate flow through the nozzle. A high R% implies that the printed structure can recover its shape rapidly and maintain structural fidelity. The LAOS‐SPP metrics comprehensively reflect both structural strength and recovery dynamics. High values of $G_{t,max}^{\prime }$ and 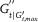 imply that the ink retains a robust structure under repeated flow‐rest cycles, contributing to both storage stability and rapid post‐extrusion recovery. 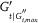 reflects the abruptness of elastic yielding, where more negative values indicate a sharper transition. This suggests that the flow initiates more rapidly upon entering the contraction channel. The accompanying parameter, 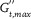, represents how quickly and intensely the structure collapses to induce flow before reaching a steady viscoplastic flow state. 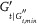 indicates the residual elastic properties of the fully fluidized ink under high‐strain‐rate conditions during extrusion. A higher value suggests that some elastic properties remain during flow, which can generate recoil‐like inward tension on the filament. This recoil may contribute to the flow retraction of the filament, thereby affecting the resolution and fidelity of fine structural details. Finally, 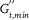 reflects the degree of shear thinning under high‐strain rates, which is directly related to the ease of flow through the nozzle. If this value is excessively low, the ink may become over‐fluidized, leading to undesirable spreading and reduced printing resolution.

**Table 1:** Meaning of Feature variables in terms of the actual 3D printing process.

Measurements	Metrics	Interpretation [Corresponding subprocess]
LAOS‐ SPP analysis	$G_{t,max}^{\prime }$/ 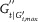	Elastic/Viscous properties of hydrogel at the most‐structured state during repeated flow and stoppage condition where strain rate approaches zero (Reservoir storage process, post‐extrusion recovery and relaxation)
	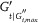 / 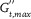	Elastic/Viscous properties when hydrogel inks initially yield through elastic yielding behavior (Initial flow through the contraction channel)
	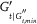 / 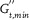	Elastic/Viscous properties when hydrogel ink is fully fluidized at high‐strain‐rate, corresponding to a viscoplastic flow state (High‐strain‐rate deformation during nozzle passage)
Strain amplitude sweep	$G_{lin}^{\prime }$/ 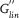	Elastic/Viscous properties under near‐equilibrium conditions reflecting fully relaxed network structure in the absence of significant deformation (Reservoir storage process, post‐extrusion recovery and relaxation)
	γ_*y*_	Strain threshold at which the hydrogel ink network begins to exhibit incipient structural breakdown, marking the onset of yielding behavior. (Post‐extrusion recovery and relaxation)
Stress amplitude sweep	σ_*y*_	Stress threshold at which the hydrogel ink network begins to exhibit incipient structural breakdown, marking the onset of yielding behavior. (Post‐extrusion recovery and relaxation)
3ITT	R%	Recovery rate of viscosity representing network rebuilding during strain rate reduction following high‐strain‐rate (Transition between the high‐strain‐rate nozzle passage and post‐extrusion recovery and relaxation)

### 2.4 Printability Quantification as a Target Variable in a Predictive Model

**Figure 4:**
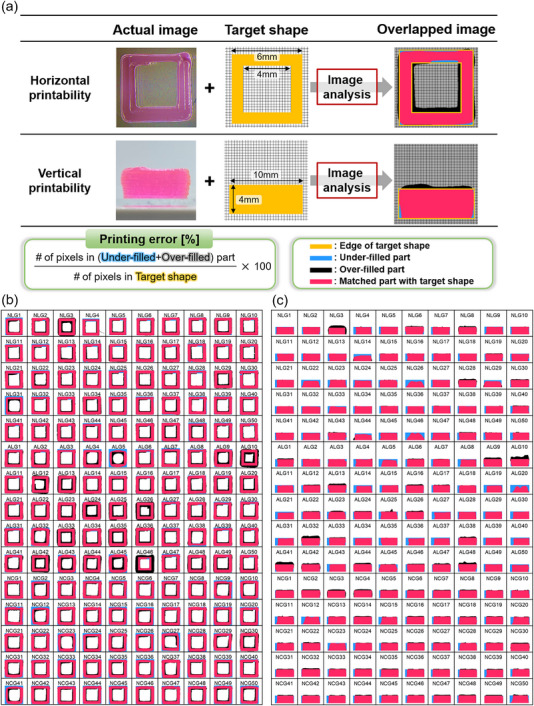
a) Quantification of horizontal and vertical printability with image analysis. Image analysis results of 3D‐printed hydrogel inks with 150 different formulations. b) Top‐view image analysis for horizontal printability quantification, c) Side‐view image analysis for vertical printability quantification.

For the quantitative analysis of the rheology‐printability correlation, we defined printability parameters—printing errors and printing inconsistency in both horizontal and vertical directions—based on image analysis of the printed hydrogel. **Figure** **4**a illustrates the calculation methods for these errors. To assess horizontal printability error, we printed an open‐square pattern (Figure 4a, first row) and captured its top‐view image under consistent conditions. The image was projected onto the target shape, identifying three distinct regions: the under‐filled area (blue), the over‐filled area (black), and the correctly matched area. The horizontal printing error, quantifying the extent of under‐ or over‐filling, was defined as the ratio of the total number of pixels in the under‐filled and over‐filled regions to the number of pixels in the target shape. This error serves as an indicator of printing performance degradation caused by the spreading or blurring of the printed hydrogel ink.

The vertical printing error was defined similarly. A cylindrical structure with a 10 mm diameter and 4 mm height, formed by stacking ten circular layers sequentially, was printed (Figure 4a, second row), and its side‐view image was captured under consistent conditions. The image was projected onto the side‐view representation of the target shape—a 10 mm × 4 mm rectangle—to identify the same three regions. The vertical printing error, calculated as the ratio of the total number of pixels in the under‐filled and over‐filled areas to those in the target shape, serves as an index of printing performance degradation due to sagging or uneven deposition.

Figure 4b,c presents the image analysis results for 150 hydrogel inks. Here, the sample names NLG, ALG, and NCG denote NIPAM‐Laponite hydrogel, Acrylamide‐Laponite hydrogel, and NIPAM‐Carbomer hydrogel, respectively. Although only one image per hydrogel ink is displayed, image analysis was performed three times for each sample to ensure reproducibility. The average printing error from these three independent printings was used as the target variable for the printing error predictive model. Additionally, the variation in printing errors across the three independent printings and image analyses, referred to as printing inconsistency, represents the variability in a hydrogel ink's 3D printing performance and was used as the target variable for the predictive model of printing process inconsistency. It should be noted that hydrogel inks remained in a pre‐gel state without exposure to ultraviolet (UV) light during the 3D‐printing and image analysis to eliminate curing effects and isolate the influence of rheological properties.

### 2.5 Random Forest Regression Model and Permutation Importance Analysis

To effectively analyze the nonlinear correlation between the rheological metrics used as feature variables and printing errors as the target variables, we employed a Random Forest (RF) regression model. The RF regression models were developed using the *RandomForestRegressor* module in *scikit‐learn*. The number of decision trees was set to a constant value of 1000 for efficient calculation, while other hyperparameters, including the maximum depth, were optimized through grid search methods. Among various machine learning techniques, RF regression was chosen for its advantages, including robustness against overfitting, resistance to outliers and data noise, and the ability to interpret feature importance.^[^ ^82^, ^83^, ^84^ ^]^ Details of the RF regression model and its constituent decision trees are provided elsewhere, and we briefly explain the workflow of our predictive model.

**Figure 5:**
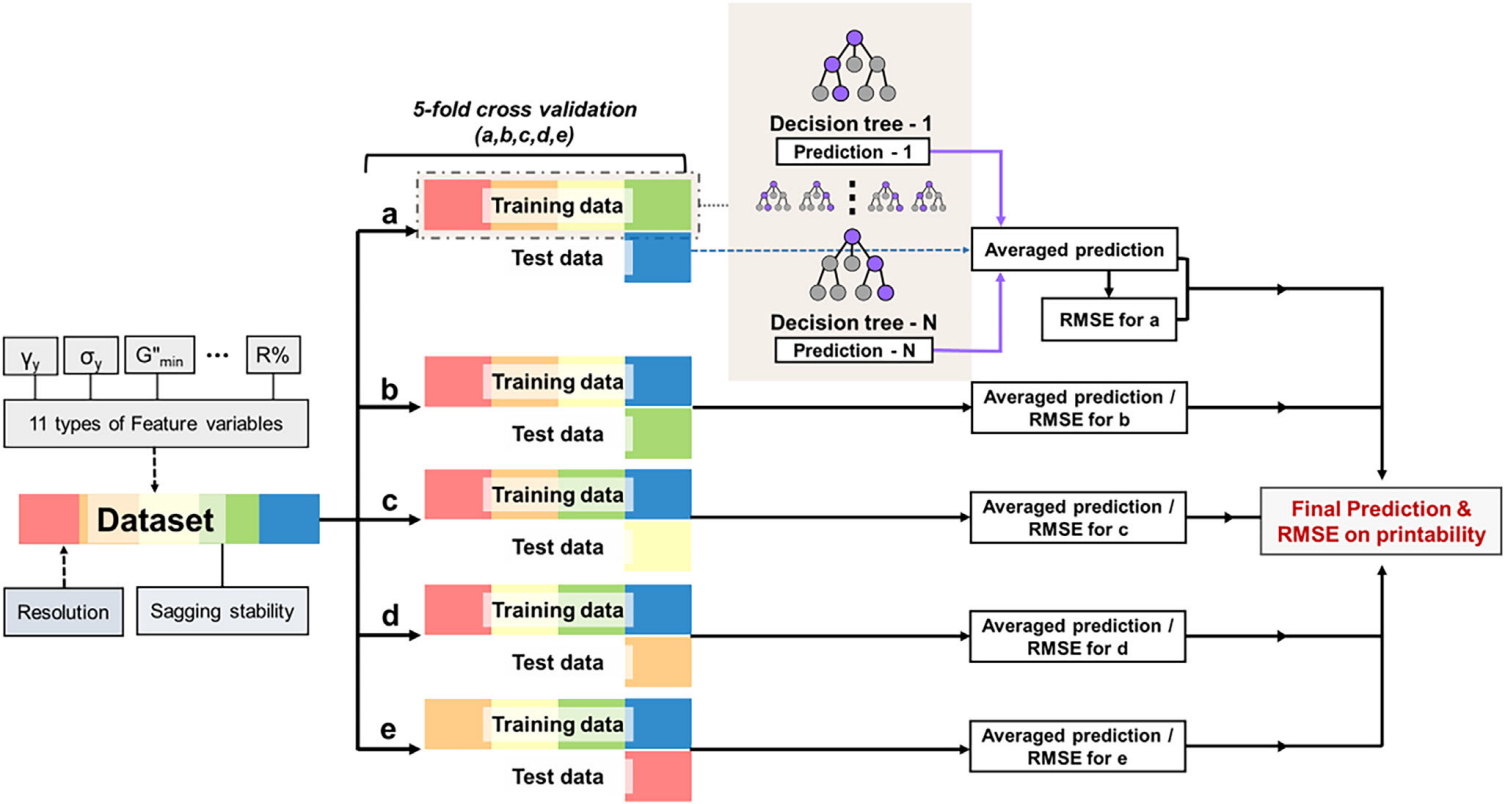
Workflow diagram of the predictive model for 3D‐printing errors, along with examples of decision trees used in the predictive model.

**Figure** **5** illustrates the workflow diagram of the predictive model for 3D‐printing printing errors in this study. To maximize the use of all data by utilizing them for both training and testing, we employed the fivefold cross‐validation technique. The RF regression model with fivefold cross‐validation works by randomly partitioning the total 150 rheology‐printability dataset into five subsets, utilizing four subsets for training and one for validation in each iteration. This process is repeated five times, ensuring that each subset serves as the validation set once.

RF is an ensemble learning method that constructs multiple decision trees during training and averages their predictions to improve accuracy and robustness. Figure S1 (Supporting Information) visualizes one of the decision trees used in the development of the predictive model for horizontal printability. As shown, each decision tree in the RF model makes predictions based on recursive binary splits of input features, selecting the most significant feature at each node to minimize prediction error. By aggregating predictions from multiple trees, RF reduces variance and prevents overfitting, leading to a more stable and reliable model.

The final model performance is evaluated in terms of the root mean squared error (RMSE) given as below,

(1) $$\mathrm{RMSE}\;=\sqrt{\frac{1}{n}\times \sum_{i=1}^{n}{\left(y_{i}-{\hat{y}}_{i}\right)}^{2}}\;$$

where *n* is the number of samples, *y_i_* represents the actual printing error, and ${\hat{y}}_{i}$ denotes the predicted printing error. The final model performance is averaged across all folds, reducing overfitting and improving generalization, while the ensemble of multiple decision trees in the RF model enhances predictive accuracy and robustness.

To assess the relative importance of each rheological metric in the predictive model, we employed a model‐agnostic technique, the permutation importance analysis. It works by measuring the decrease in model performance when the values of a rheological metric are randomly shuffled, thereby breaking the relationship between that rheological feature and the printing error. The underlying principle is that if a rheological feature is important for making predictions, disrupting its values should significantly degrade the model's accuracy. The process involves first computing the baseline performance of the trained model, represented by RMSE in this work. Then, for each rheological feature, its values are permuted while keeping other features unchanged, and the model's performance is re‐evaluated. The difference between the baseline performance and the permuted performance quantifies the importance of the rheological feature—the larger the drop in performance, the more influential the rheological feature is in the model's predictions. Although the inherent complexity of the relationship between rheological features and printability prevents an explicit conclusion, permutation importance analysis offers meaningful insights into the key rheological factors influencing printability.

### 2.6 Prediction of Horizontal Printability and Correlation Analysis with Rheological Properties

**Figure 6:**
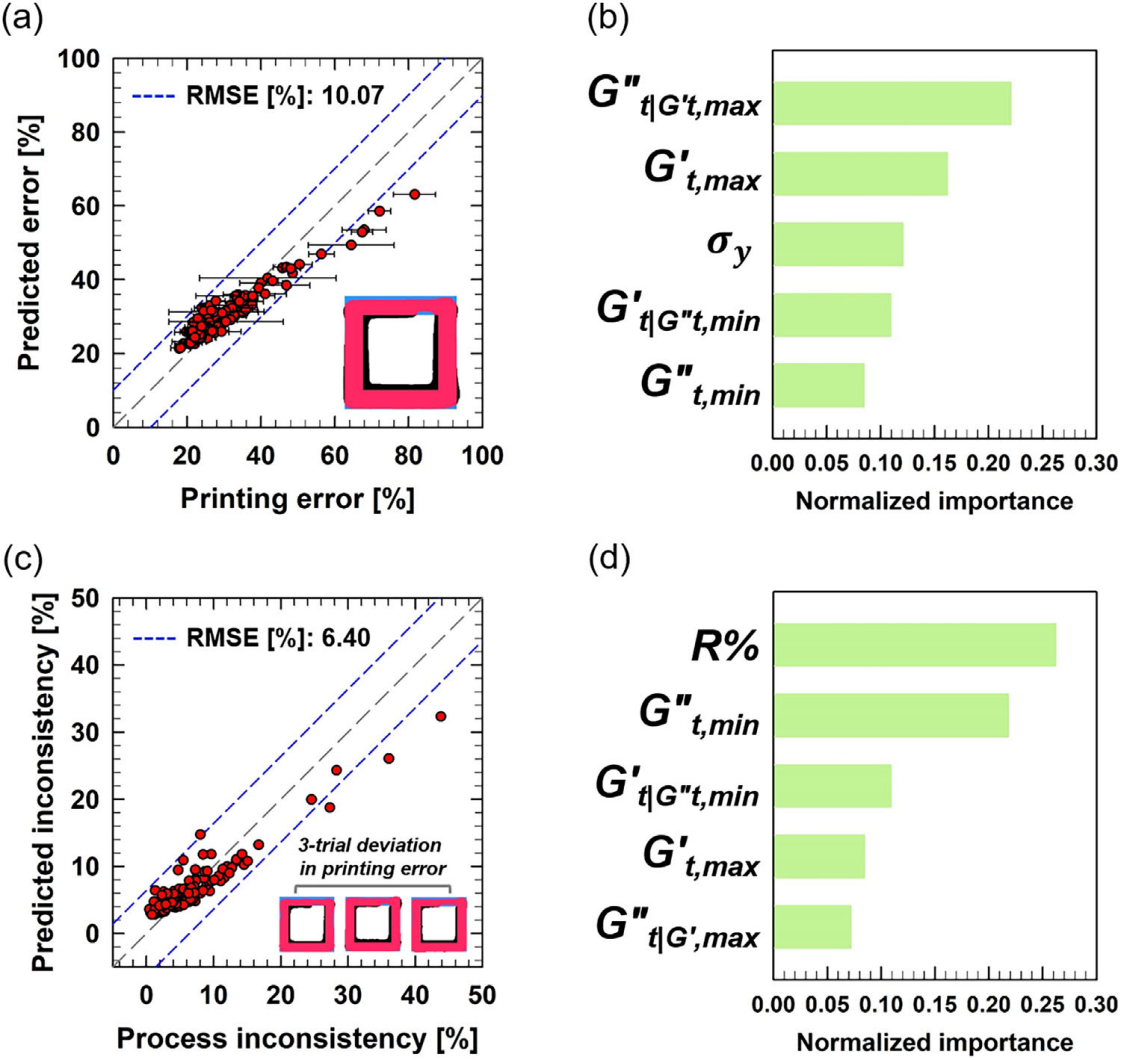
Prediction performance and permutation importance analysis for horizontal printing error and printing process inconsistency. a) Prediction performance of the model for horizontal printing error. The blue short‐dashed line indicates the RMSE deviation range, while the gray long‐dashed line represents the accurate prediction line (***y *** = *** x***). The error bars denote deviations from the average value obtained through three measurements. b) The top five features with the highest permutation importance in the predictive model for horizontal printability. c) Prediction performance of the model for printing inconsistency in the horizontal direction. d) The top five features with the highest permutation importance in the predictive model for printing inconsistency in the horizontal direction.


**Figure** **6** presents the performance and permutation importance analysis results of the predictive model for printing error and printing process inconsistency in the horizontal direction. The model demonstrates remarkable predictive capability, achieving RMSE values of 10.07% for printing error and 6.4% for printing inconsistency, as shown in Figure 6a,c. The permutation importance analysis in Figure 6b,d reveals the most important five rheological features in determining the horizontal printing error and printing inconsistency, respectively. Intriguingly, of the five most important rheological features, four are derived from LAOS‐SPP analysis for both cases of the printing error and printing inconsistency, highlighting the effectiveness of our strategic approach based on the analogy between LAOS and 3D‐printing flow.

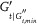
 and 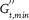, ranked as the fourth and fifth most important features for predicting horizontal printing error in Figure 6b, are associated with the fully fluidized phase occurring during high‐strain‐rate deformation in the nozzle passage, marked by the blue circle in Figure 2. The first and second most important features—
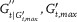
—correspond to the most structured state during post‐extrusion recovery and relaxation process, represented by the yellow square in Figure 2. The significant importance of these four rheological features, derived from the two sequential points marked by the blue circle and yellow square, underscores the critical role of the rheological transition from high‐strain‐rate deformation during nozzle passage to post‐extrusion recovery and relaxation, highlighted by the green lines in Figure 2, in determining the horizontal printing error. Specifically, the remarkably high importance of 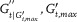 signifies that printability is closely related to how quickly and to what extent the hydrogel inks recover their viscoelastic properties after transitioning from the fluidized phase—where rheological properties are represented by 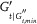 and 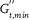
—during the repeated flow and cessation cycles in the 3D printing process. In a similar vein, aside from the four LAOS‐SPP metrics, σ_*y*_ was identified as the third most important feature, highlighting the significance of the maximum stress that the printed hydrogel can withstand following the extrusion and recovery process.

Our result reveals that the rheological transition from high‐strain‐rate deformation during nozzle passage to post‐extrusion recovery and relaxation also plays a critical role in the determination of horizontal printing inconsistency. The permutation importance analysis for the horizontal printing inconsistency illustrated in Figure 6d demonstrates that the transient rheological properties of 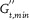 and 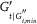 at the fully fluidized phase occurring during high‐strain‐rate deformation in the nozzle passage are the second and third most important features, followed by the $G_{t,max}^{\prime }$ and 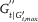 at the most structured state in the recovery and relaxation process after extrusion.

While the remarkable importance of the four LAOS‐SPP metrics coincides with the horizontal printing error case, the printing inconsistency is more influenced by the high‐strain‐rate deformation during the nozzle passage process and relevant rheological properties at the fluidized state of hydrogel inks. Contrary to the horizontal printing error case for which 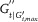 and $G_{t,max}^{\prime }$ corresponding to the post‐extrusion recovery process have higher importance than 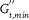 and 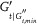, the horizontal printing inconsistency is shown to be more influenced by 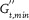 and 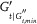 that are related to the high‐strain‐rate deformation in the nozzle process. Moreover, R% that represents the recovery rate of viscosity when the strain rate is reduced during flowing is shown to be the feature of the highest importance, while the yield stress (σ_*y*_) related to the post‐extrusion recovery process, which was ranked as the third most important feature for the horizontal printing error, is not included in the top five important features. Such differences imply that while the horizontal printing error is more closely related to the rheological properties at more solid‐like states, after post‐extrusion recovery and relaxation process, the printing inconsistency (or printing reproducibility) is more affected by the rheological properties at more fluid‐like states, the high‐strain‐rate deformation and flow in the nozzle.

### 2.7 Prediction of Vertical Printability and Correlation Analysis with Rheological Properties

**Figure 7:**
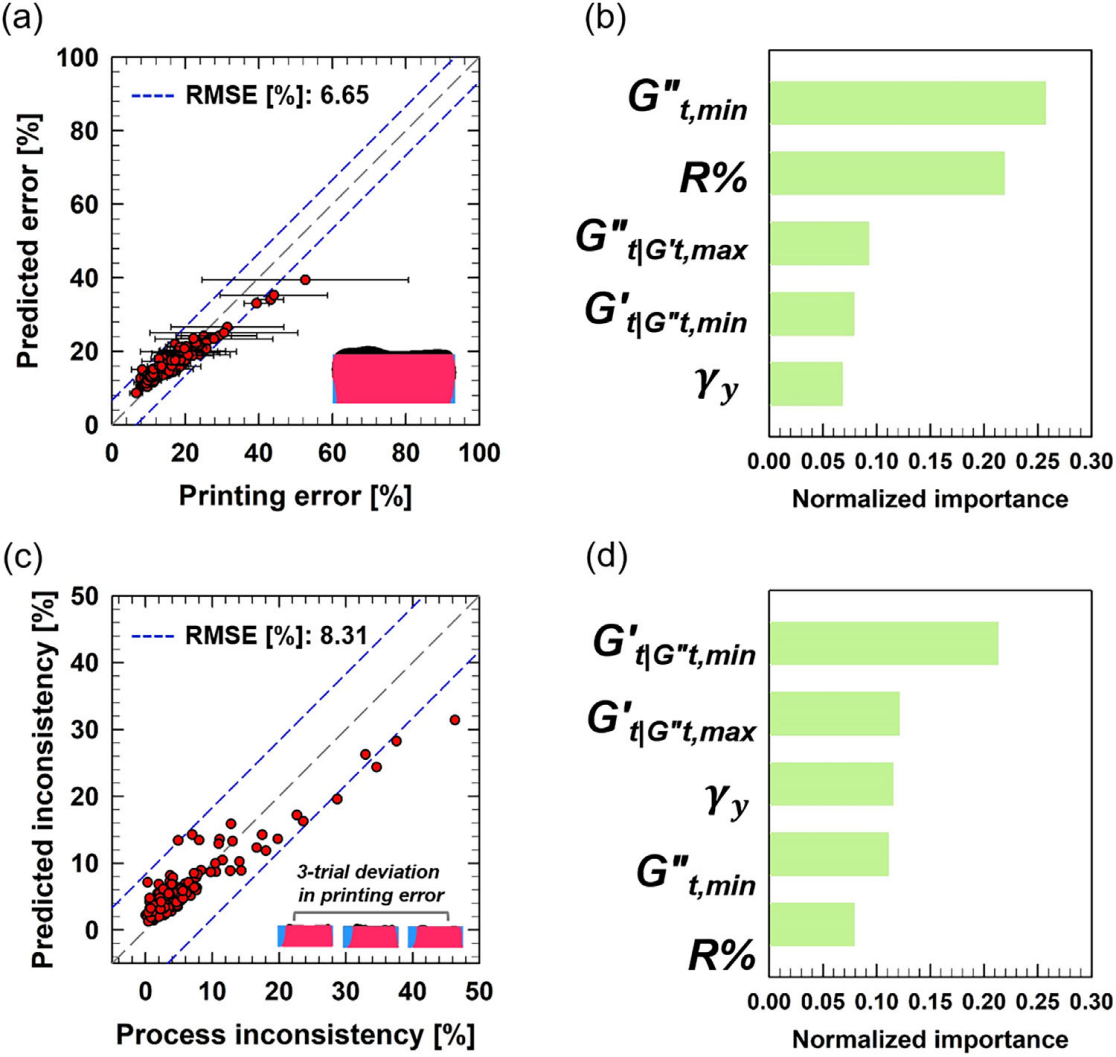
Prediction performance and permutation importance analysis for vertical printing error and printing process inconsistency. a) Prediction performance of the model for vertical printing error. The blue short‐dashed line indicates the RMSE deviation range, while the gray long‐dashed line represents the accurate prediction line (*y*  =  *x*). The error bars denote deviations from the average value obtained through three measurements. b) The top five features with the highest permutation importance in the predictive model for vertical printability. c) Prediction performance of the model for printing inconsistency in the vertical direction. d) The top five features with the highest permutation importance in the predictive model for printing inconsistency in the vertical direction.

The performance and permutation importance analysis results of the predictive model for printing error and printing process inconsistency in the vertical direction are presented in **Figure** **7**. The model achieves RMSE values of 6.65% for printing error prediction and 8.31% for printing inconsistency prediction, as shown in Figure 7a,c, indicating reasonable predictive accuracy for vertical printability.

In determining the vertical printing error, the viscous properties of the hydrogel inks were found to key contributors, as shown in Figure 7b. Specifically, 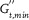 and R% were ranked as the first and second most important features, respectively. The highest importance of 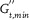 highlights the critical role of the viscous behavior of the hydrogel ink in its fully fluidized state during high‐strain‐rate flow through the nozzle. Likewise, the importance of R%—which reflects the shear hysteresis of viscous properties, or the rate at which the ink adapts its viscosity to changing flow conditions—indicates its substantial contribution to vertical printing error.

Intriguingly, while the horizontal printability was found to the more influenced by rheological properties associated with the post‐extrusion recovery and relaxation processes (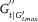, $G_{t,max}^{\prime }$, and σ_*y*_), the vertical printability was predominantly governed by properties linked to high‐strain‐rate flow within the nozzle (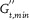, R%, and 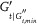). This distinction suggests that vertical printability is primarily determined during the nozzle passage, whereas horizontal printability is shaped during the recovery and relaxation phase following extrusion.

The third most important feature 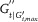 can be discussed in conjunction with the first important feature, 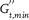. As previously noted, 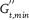 and 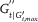 correspond to two sequential points, marked by the blue circle and yellow square in Figure 2, representing the transition in viscous behavior from the fully fluidized state within the nozzle to the more structured state after extrusion. The significant importance of both features underscores the critical role of transient viscous properties that bridge the high‐strain‐rate nozzle flow and the post‐extrusion recovery process.

Printing inconsistency in the vertical direction differs from previous cases—such as horizontal printability and vertical printability—which were primarily determined by the transition between the fully fluidized state in the nozzle and the post‐extrusion recovery on the substrate. The vertical printing inconsistency appears to be more significantly influenced by the rheological transition between the partially yielded and fully fluidized states. Two transient elastic moduli, 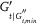 and 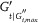
—representing elastic properties at the partially yielded and fully fluidized states, and marked by the blue circle and red star in Figure 2, respectively—were identified as the first and second most important features in the permutation importance analysis (Figure 7d).

**Figure 8:**
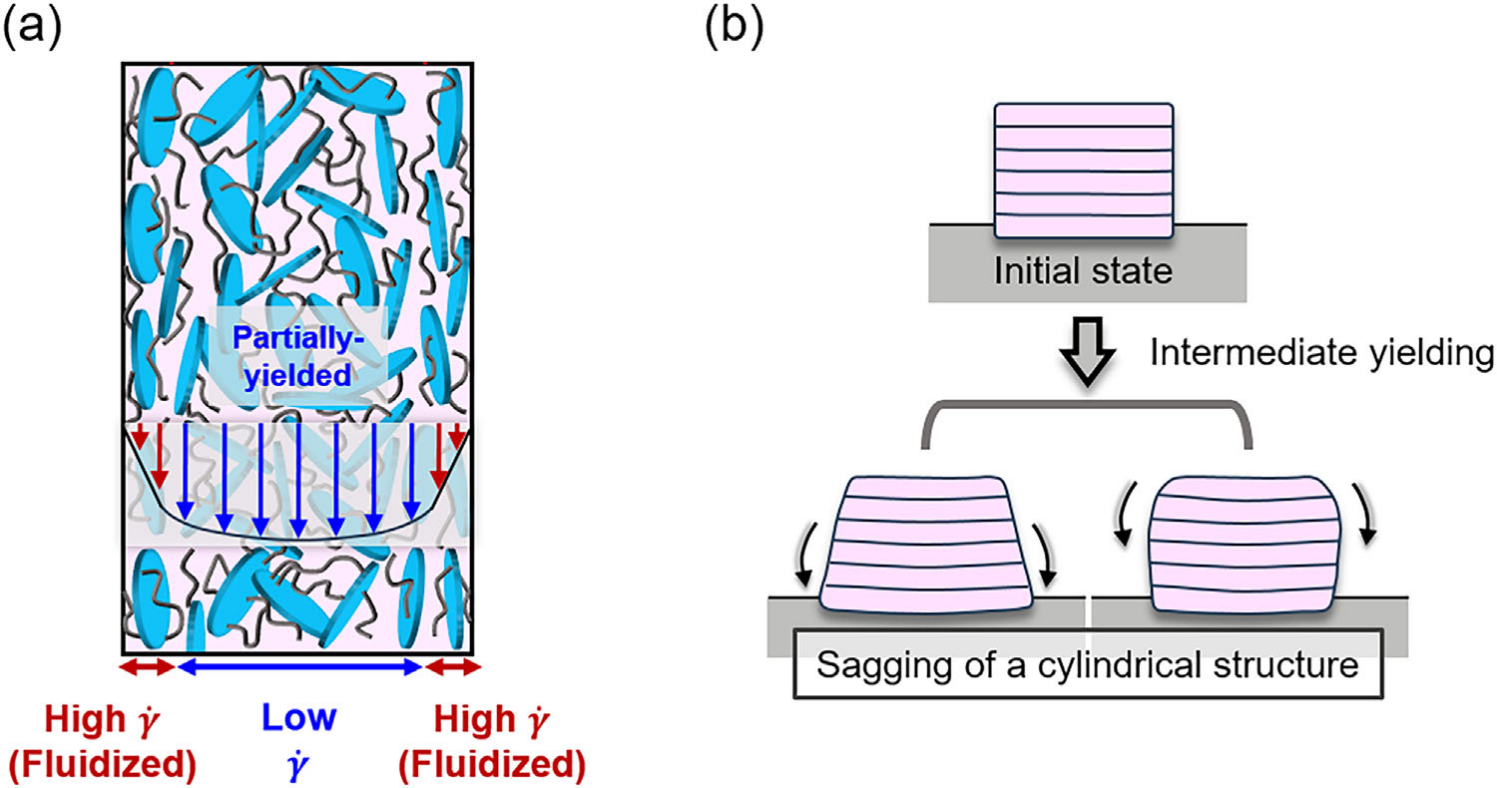
Shear‐induced heterogeneity and sagging behavior influence printing inconsistency in the vertical direction. a) Strain rate distribution and incomplete fluidization of the hydrogel ink's structure inside the nozzle. b) Initial and post‐extrusion states of a printed cylindrical hydrogel structure. The top image shows the initial printed shape immediately after extrusion, while the bottom images illustrate the sagging‐induced deformation.

Although the exact correlation between printing inconsistency and these two transient elastic moduli remains unclear, several potential interpretations can be proposed from rheological and processing perspectives. The significance of these moduli may indicate that the incompletely fluidized hydrogel network, which retains elastic properties after initial yielding, plays a critical role in the emergence of inconsistency. Ideally, this network is expected to be fully fluidized under the high‐strain‐rate flow through the nozzle. However, in the presence of complex flow phenomena such as shear banding or shear‐induced heterogeneity,^[^ ^85^, ^86^, ^87^ ^]^ parts of the network may pass through the nozzle without complete fluidization and be deposited onto the substrate, and one possible case of this phenomenon is schematically illustrated in **Figure** **8**a. In particular, it highlights that the shear rate is lower at the center of the nozzle than near the wall, allowing parts of the hydrogel ink's network structure to remain partially intact and be extruded without complete fluidization, while the hydrogel ink near the wall undergoes full structural breakdown due to the higher shear rate. It is plausible that such uncontrollably heterogeneous flow of residual network chains contributes to inconsistent deposition and, consequently, vertical printing inconsistency.

Another possible interpretation involves the sagging of hydrogel ink after deposition, as shown in Figure 8b. Following high‐strain‐rate flow in the nozzle, the deposited hydrogel begins to relax and recover its intrinsic elastic and viscous properties. As additional layers are printed, the accumulated weight imposes stress that may exceed the hydrogel ink's yield strain or yield stress threshold, leading to localized yielding and sagging. Distinguished from the rapid deformation inside the nozzle, sagging occurs more gradually under low‐strain‐rate conditions, which correspond to the intermediate yielding regime between initial yielding and full fluidization. As can also be observed in Figure 4c, this sagging does not cause complete collapse but results in bulging of either the bottom or top part of the cylindrical structure. The strong correlation between 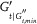 and 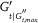
—which represents elastic behavior in this intermediate yielding behavior— and vertical printing inconsistency suggests that the variability in vertical printability is primarily governed by this sagging behavior. This stands in contrast to the mechanism underlying vertical printing error, which is predominantly governed by the viscous properties of the hydrogel ink in its fully fluidized state. The third most important feature, the yield strain (γ_*y*_), around which the intermediate yielding occurs, further reinforces the interpretation provided by 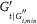 and 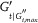.

As an additional approach to assess the generalization capability of the predictive model for new compositions, an independent validation was conducted using the existing dataset. The full dataset was randomly divided into 70% for model training and 30% for validation. The model was trained on the training set using fivefold cross‐validation, and its predictive performance was subsequently evaluated on the independent validation set. As presented in Figure S2 (Supporting Information), the results of the independent validation support that the developed model and its physical interpretations can be reliably extended to new formulations.

## 3 Conclusion

This study presents a comprehensive data‐driven framework for elucidating the intricate relationship between the rheological behavior of hydrogel inks and their 3D printability. By leveraging advanced rheological metrics—specifically those derived from large‐amplitude oscillatory shear (LAOS) stress decomposition—and integrating them with machine learning, we accurately predicted and mechanistically interpreted printability metrics, including horizontal and vertical printability and structural inconsistency. These rheological descriptors effectively capture the nonlinear, transient flow behavior under realistic 3D printing conditions, distinguishing this work from previous studies and offering a powerful methodology for process‐property analysis in soft material printing.

This study demonstrates that 3D printability of hydrogel inks—quantified via image‐based metrics for horizontal and vertical printing errors and inconsistencies—can be reliably predicted using rheological metrics derived under realistic printing conditions. The RF regression models achieve high accuracy (RMSE ≤ 10%), and permutation‐based feature importance analysis reveals that most of the influential rheological metrics are derived from LAOS‐SPP analysis. Horizontal printability is predominantly influenced by rheological properties associated with the post‐extrusion recovery and relaxation process, indicating the significance of solid‐like behavior after flow cessation. In contrast, horizontal inconsistency is more strongly influenced by the fully fluidized state within the nozzle. Vertical printability is mainly affected by viscous responses during high‐strain‐rate flow, while vertical inconsistency is linked to elastic responses associated with incomplete fluidization and sagging under partially yielded conditions. Collectively, these findings underscore that distinct flow regimes within the 3D printing process govern different aspects of printability. These regime‐specific influences are difficult to capture using conventional rheological assessments, underscoring the distinct advantage of LAOS‐SPP in reflecting the actual printing dynamics. Incorporating transient, nonlinear rheological descriptors thus enables meaningful, physics‐informed predictions of printing behavior across a wide range of hydrogel formulations.

Importantly, this study serves as a representative example of how process characteristics can be effectively analyzed based on rheological behaviors that faithfully reflect actual fluid processing conditions. It emphasizes the value of rheology‐informed descriptors as meaningful and transferable parameters in evaluating and optimizing printing outcomes—thus demonstrating both the conceptual significance and practical utility of the proposed framework. Moreover, while the predictive model developed in this study was trained on specific hydrogel formulations, its generalization to other material systems is theoretically plausible if similar flow conditions—such as the repeated flow and rest cycles observed in DIW printing—exist and the same set of rheological measurements, including LAOS, can be conducted. However, in practical applications, printing conditions such as nozzle size, flow rate, temperature, and humidity can vary depending on the system and environment. These parameters influence not only the printing process itself but also the rheological conditions experienced by the ink. Therefore, to ensure the applicability of the model, it is critical to appropriately adjust rheological testing protocols to reflect the actual flow conditions during printing. For instance, the LAOS test allows flexible adjustment of test conditions, making it well‐suited to accommodate such variations. The strain amplitude can be set to match the maximum strain rate encountered during extrusion, while the frequency can be tuned to reflect the specific flow–rest cycle of the given printing system. In addition, it is essential to identify and accurately collect rheological features that reflect these adjusted testing conditions.

While this study aimed to elucidate the relationship between rheological behavior and 3D printability under fixed printing conditions, it is important to recognize that printability is governed not only by material properties but also by printing parameters. Ultimately, identifying the optimal combinations of material properties and printing parameters is essential for achieving reliable printing outcomes. In future studies, the framework proposed in this work may be further expanded by constructing a more comprehensive dataset that incorporates variations in both ink composition and printing conditions, enabling broader generalization and application across diverse material systems.

From a broader perspective, the findings of this study provide essential guidelines for the design of hydrogel materials in soft robotics and stretchable electronics. By enabling precise prediction and control of printability from rheological characteristics alone, the framework supports the development of sustainable and functional materials tailored for advanced additive manufacturing. This underscores the critical role of rheology‐based design strategies in advancing next‐generation materials and processing technologies with sustainability at the forefront.

## 4 Experimental Section

### Preparation of Hydrogel Inks

Three types of hydrogel inks were prepared, totaling 150 samples (50 samples each), based on protocols adapted from previous studies:^[^ ^18^, ^67^, ^68^ ^]^ two Laponite‐based hydrogel inks 1) AAm‐Laponite hydrogel, 2) NIPAM‐Laponite hydrogel, and one Carbomer‐based hydrogel ink 3) NIPAM‐Carbomer hydrogel. Rhodamine 6G was used as a dye in all hydrogel inks. To prepare AAm‐based hydrogel ink, AAm, *N*‐*N*‘methylenebisacrylamide (BIS), laponite, and Irgacure 2959 were added to distilled (DI) water and stirred for 24 h using a magnetic stirrer (PC‐420D, Corning). Laponite‐NIPAM hydrogel ink was prepared by stirring NIPAM, poly *N*‐isopropyl acrylamide (pNIPAM), laponite, and Irgacure 2959 for 5 min using a magnetic stirrer. DI water was then added, and the mixture was stirred for an additional 24 h. To prepare Carbomer‐NIPAM hydrogel ink, NIPAM, α‐ketoglutaric acid (α‐keto), BIS, and NaOH were dissolved in water to make 2, 0.1, 0.1, and 10 M aqueous solutions, respectively. The NIPAM solution, α‐keto solution, and BIS solution were stirred on a magnetic stirrer for 5 min, and Carbomer 940 powder was added and stirred for an additional 2 h. NaOH solution was added to this uniformly mixed solution. The compositions of all hydrogel inks prepared are listed in **Table** **2**, and the detailed compositions for each type of hydrogel ink are provided in Supporting Information Tables S1–S3 (Supporting Information).

**Table 2:** Composition information of the three types of hydrogel inks used.

Material [wt%]	Type 1	Type 2	Type 3
Laponite	5.4–10.7	5.2–9.4	–
AAm	4.8–11.1	–	–
BIS	1.0–2.8	–	0.002–0.015
Irgacure	0.4–1.3	0.1–0.3	–
Carbomer	–	–	0.4–2.3
NIPAM	–	5.2–11.4	11‐18
α–keto	–	–	0.01–0.07
NaOH	–	–	0.08‐0.48
pNIPAM	–	0.1–0.5	–
DI water	78–86	81–88	80–86

### Measurement of Rheological Properties

Rheological analysis of 150 hydrogel inks was conducted using a stress‐controlled rheometer (HR‐20; TA instruments). A 40 mm parallel plate with a sandblasted geometry was used for measurements to avoid potential wall‐slip effects. Prior to rheological tests, to ensure that all samples were uniformly preconditioned and unaffected by any previously accumulated strain, they were pre‐sheared at a strain of 10 for 300 s and then allowed to relax for 180 s. Dynamic strain and stress amplitude sweep tests were performed at a frequency of 1 rad s^−1^. Three‐interval thixotropy tests (3ITT) were conducted in the following order: strain rate of 0.1 s^−1^ for 120 s, 100 s^−1^ for 10 s, and 0.1 s^−1^ for 120 s.

### Sequence of Physical Processes (SPP) Analysis

Like most materials, the stress response of hydrogel inks under oscillatory deformation was a combination of elastic contributions related to the applied strain and viscous contributions related to the applied strain rate, analyzed through the SPP scheme. Thus, the stress response was considered a function of strain (γ) and dimensionless strain rate ($\dot{\gamma }/\omega$), represented as $\sigma (\gamma ,\frac{\dot{\gamma }}{\omega })$, and depicted as a trajectory of the strain (γ)‐strain rate($\dot{\gamma }/\omega )$‐stress(σ) in Figure 2f as the solid sky‐blue line. The instantaneous rheological state at a specific time (*t*) was expressed as a position vector $\vec{R}\;\left(t\right)=\left(\gamma \left(t\right),\frac{\dot{\gamma }\left(t\right)}{\omega },\sigma \left(t\right)\right)$, on the 3D trajectory. The SPP scheme employs the Frenet‐Serret apparatus as a mathematical tool to discuss the characteristics of specific points on the trajectory, which includes the Frenet‐Serret frame composed of tangent ($\vec{T}$), normal ($\vec{N}$), and binormal ($\vec{B}$) vectors, along with two scalars (curvature and torsion). Each vector is defined as follows:

(2) $$\vec{T}\left(t\right)=\frac{\vec{R^{\prime }}\left(t\right)}{\left|\vec{R^{\prime }}\left(t\right)\right|},\;\;\vec{N}\left(t\right)={\frac{\vec{T^{\prime }}\left(t\right)}{\left|\vec{T^{\prime }}\left(t\right)\right|}}^{\prime },\;\;\vec{B}\left(t\right)=\vec{T}\left(t\right)\times \vec{N}\left(t\right)$$

The tangent vector ($\vec{T}\left(t\right)$) is defined as the unit vector tangent to the direction of motion, and the normal vector ($\vec{N}\left(t\right)$) is defined as the unit vector pointing in the direction of the time derivative of the tangent vector. The binormal vector ($\vec{B}\left(t\right)$), defined as the cross product of the tangent and normal vectors, was thus orthogonal to both. As shown in Figure 2f, the position vector moves from *t*_1_ to *t*_2_ over time, adjusting the Frenet‐Serret frame accordingly. Since the tangent and normal vectors indicate the directionality and change in direction of the trajectory, they can form the osculating plane, the plane in which the curve sits in locally. According to the Frenet–Serret equation, the binormal vector was orthogonal to the osculating plane. Therefore, the normal vector of the osculating plane at $\vec{R}\left(t\right)$ is given as the binormal vector.

(3) $$\begin{array}{c} B_{\gamma }\left(t_{1}\right)\left(\gamma \left(t\right)-\gamma \left(t_{1}\right)\right)+B_{\frac{\dot{\gamma }}{\omega }}\left(t_{1}\right)\left(\frac{\dot{\gamma }}{\omega }\left(t\right)-\frac{\dot{\gamma }}{\omega }\left(t_{1}\right)\right)\\ +B_{\sigma }\left(t_{1}\right)\;\left(\sigma \left(t\right)-\sigma \left(t_{1}\right)\right)=0 \end{array}$$

As each term's *t* is replaced with *t*_1_ ± Δ*t*, each variable incorporates the value at the reference point *t*_1_ along with a small amount of variation near it. This substitution allows Equation (3) to be expressed in differential form as follows:

(4) $$\begin{array}{c} B_{\gamma }\left(\gamma \left(tt_{1}\pm \Delta t\right)-\gamma \left(t_{1}\right)\right)+B_{\frac{\dot{\gamma }}{\omega }}\left(t_{1}\right)\left(\frac{\dot{\gamma }}{\omega }\left(t_{1}\pm \Delta t\right)-\frac{\dot{\gamma }}{\omega }\left(t_{1}\right)\right)\\ +B_{\sigma }\left(t_{1}\right)\;\left(\sigma \left(t_{1}\pm \Delta t\right)-\sigma \left(t_{1}\right)\right)=0 \end{array}$$

(5) $$B_{\gamma }d\gamma +B_{\frac{\dot{\gamma }}{\omega }}d\left(\frac{\dot{\gamma }}{\omega }\right)+B_{\sigma }d\sigma =0$$

(6) $$d\sigma =-\frac{B_{\gamma }}{B_{\sigma }}d\gamma -\frac{B_{\frac{\dot{\gamma }}{\omega }}}{B_{\sigma }}d\left(\frac{\dot{\gamma }}{\omega }\right)$$

Equation (7) represents the total derivative of the stress $\sigma (\gamma ,\frac{\dot{\gamma }}{\omega })$.

(7) $$d\sigma =\frac{\partial \sigma }{\partial \gamma }\;d\gamma +\frac{\partial \sigma }{\partial \frac{\dot{\gamma }}{\omega }}d\left(\frac{\dot{\gamma }}{\omega }\right)$$

By comparing this with Equation (6), the transient elastic modulus ($G_{t}^{\prime }$) and transient viscous modulus (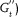 can be defined as:

(8)

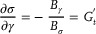

(9) $$\;\frac{\partial \sigma }{\partial \left(\frac{\dot{\gamma }}{\omega }\right)}=-\;\frac{B_{\dot{\gamma }/\omega }}{B_{\sigma }}=G_{t}^{\prime \prime }\;$$

They reflect the transient impacts of strain and strain rate on stress response, and by plotting them as shown in Figure 2g, the rheological transition behavior of materials was analyzed under large amplitude oscillatory shear (LAOS).

### 3D Printing Conditions and Effective Strain Rate

A direct ink writing (DIW) 3D printer (Inkredible+ 3D Bioprinter, Cellink) was used for 3D printing of all hydrogel inks. Cartridges containing Laponite‐based or Carbomer‐based hydrogel inks were inserted into the 3D printer. The printability of hydrogel inks can be influenced by printing parameters and environmental factors. In order to focus solely on the influence of compositional variations and to prevent printing parameters from acting as additional variables, the printing conditions were kept as consistent as possible throughout the experiments. The structures were printed on slide glass at room temperature, using appropriate pressure and nozzle sizes based on the fluidity of the material and the required printing precision. The printing pressure ranged from 10 to 230 kPa, and the nozzles used were 22G (410 µm in diameter) and 27G (200 µm in diameter). The volumetric flow rate of all hydrogel inks was maintained at ≈1.9×10^−3^ mL s^−1^, with the printing of each structure completed within 4 s for the open‐square pattern and 170 s for the cylindrical structure. The position of the printer head and the distance between the nozzle and the slide glass were calibrated to the same settings for all samples prior to printing the target structures designed with computer‐aided design (CAD).

Additionally, the effective strain rate was calculated as the hydrogel ink passed through the nozzle to determine the strain rate range for the 3ITT (Three‐Interval Thixotropy Test). Initially, the volumetric flow rate was calculated based on the volume of the printing pattern and the time required for printing. This volumetric flow rate was then divided by the nozzle's cross‐sectional area to determine the average flow velocity, which was subsequently divided by the nozzle's radius to calculate the effective strain rate. It is confirmed that the effective strain rate in the nozzle ranged from tens to several hundred s^−1^. Based on the calculated effective strain rate, a strain rate of 100 s^−1^ in the 3ITT test was applied to mimic the shear conditions experienced by the hydrogel ink passing through the nozzle. To simulate the conditions of the ink in the reservoir or after extrusion, a low strain rate of 0.1 s^−1^ was set to evaluate the recovery characteristics of the inks.

### Image Analysis

A miniature studio was constructed using an LED photo box (PULUZ, China) to standardize lighting conditions during image acquisition and ensure reproducibility across all measurements. All images were acquired using identical camera settings. The image analysis in this study was conducted in a Python environment, utilizing libraries such as *OpenCV*, *PIL*, *numpy*, and *rembg*. The proposed analysis pipeline applied identical preprocessing procedures and parameters across the entire dataset, and most processes were automated to ensure consistency across repeated experiments. First, the original images were automatically cropped to the same region using *OpenCV*. Background removal was performed using the *rembg* library, which employs a pre‐trained deep learning segmentation model to classify each pixel as printed structure or background. Pixels identified as background were processed to be transparent and were subsequently converted to black during RGB transformation. Additional color‐based filtering was performed using *PIL* to extract the structure region by selecting pixels within a specified color range. Based on the extracted coordinates, black pixels were assigned to the corresponding locations to represent the structure region, following the initialization of a new image with a white background. The resulting images were then binarized, with a threshold value of 70 uniformly applied across all samples. This threshold was optimized through preliminary testing to account for minor brightness variations remaining after background removal and color filtering. Owing to the high contrast between the printed structure containing Rhodamine 6G and the background, the binarization results were largely insensitive to small variations in the threshold value within a range near 70. Therefore, a threshold of 70 was selected to ensure robustness and minimize sensitivity to binarization boundaries. Grayscale pixel values equal to or below 70 were converted to black (0), while values above 70 were converted to white (255).

Subsequently, the reference and experimental images were compared to evaluate the printability of the printed structures. The reference image was generated as a fully black image of the same size, representing an ideal printed structure. The reference image was aligned to the bottom of the experimental image, and the best matching position was determined by shifting along both the horizontal and vertical directions to maximize the number of overlapping black pixels. Based on this alignment, unfilled and overfilled regions were quantified. The unfilled region was defined as the number of pixels that were black in the reference image but white in the experimental image, while the overfilled region was defined as the number of black pixels detected outside the reference image in the experimental image. The total printing error was quantified as the sum of unfilled and overfilled pixels normalized by the total number of black pixels in the reference image. As shown in Figure 4b,c, the final results were visualized, with matched regions displayed in pink, unfilled regions in blue, and overfilled regions in black.

## Conflict of Interest

The authors declare no conflict of interest.

## Supporting information



Supporting Information

## Data Availability

The data that support the findings of this study are available from the corresponding author upon reasonable request.
