# Supplementary material for: Data‐Driven Printability Modeling of Hydrogels for Precise Direct Ink Writing Based on Rheological Properties
Source: Adv Sci (Weinh). 2025 Jul 3;13(15):e07639. doi: 10.1002/advs.202507639 (PMC13042410; doi:10.1002/advs.202507639)
Supplement: Supplementary file 1 — Supporting Information [file ADVS-13-e07639-s001.docx]

**Supporting Information**

**Data-driven Printability Modeling of Hydrogels for Precise Direct Ink Writing Based on Rheological Properties**

Eun Hui Jeong^1^, Ji Ho Choi^2^, Han Bi Park^2^, Ji Woo Lee^2^, Seo Yeon Bae^2^, Byoung Soo Kim^3^, ChangKyu Yoon^2,4,*^, Jun Dong Park^1,4,*^


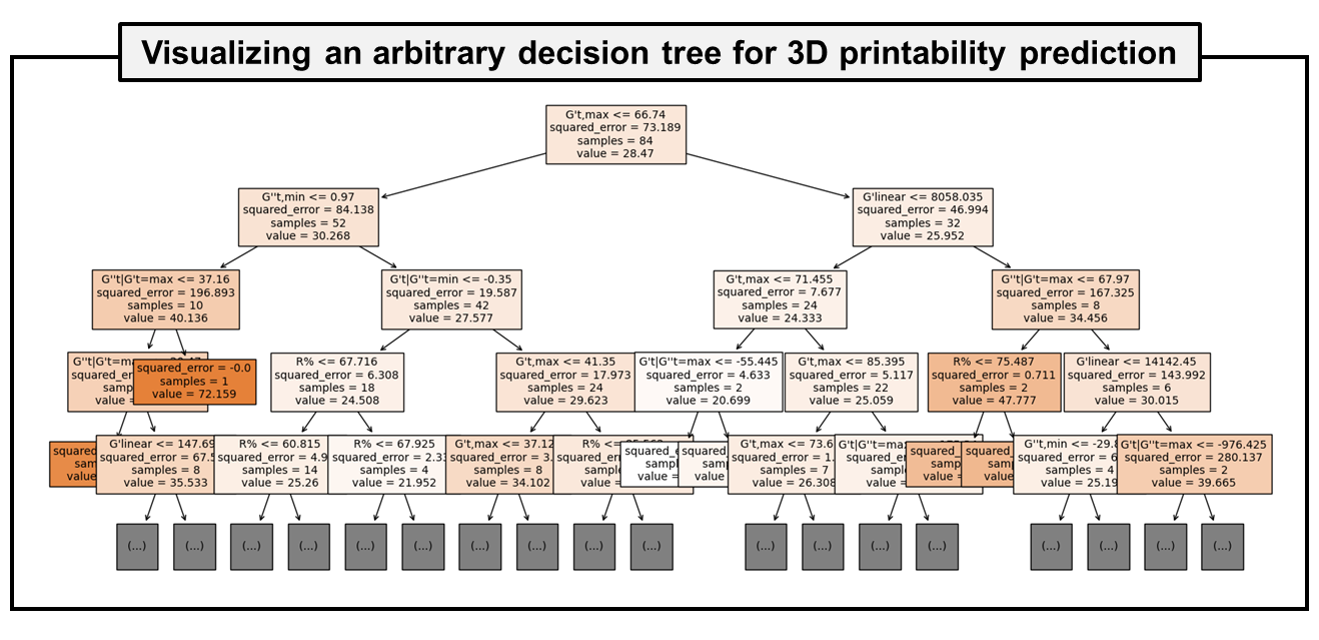


Figure S1. Visualization of an example decision tree extracted from the predictive model for horizontal printability. The tree starts from the root node and splits samples based on feature thresholds. The splitting continues until reaching leaf nodes, which assign the predicted horizontal printability values.


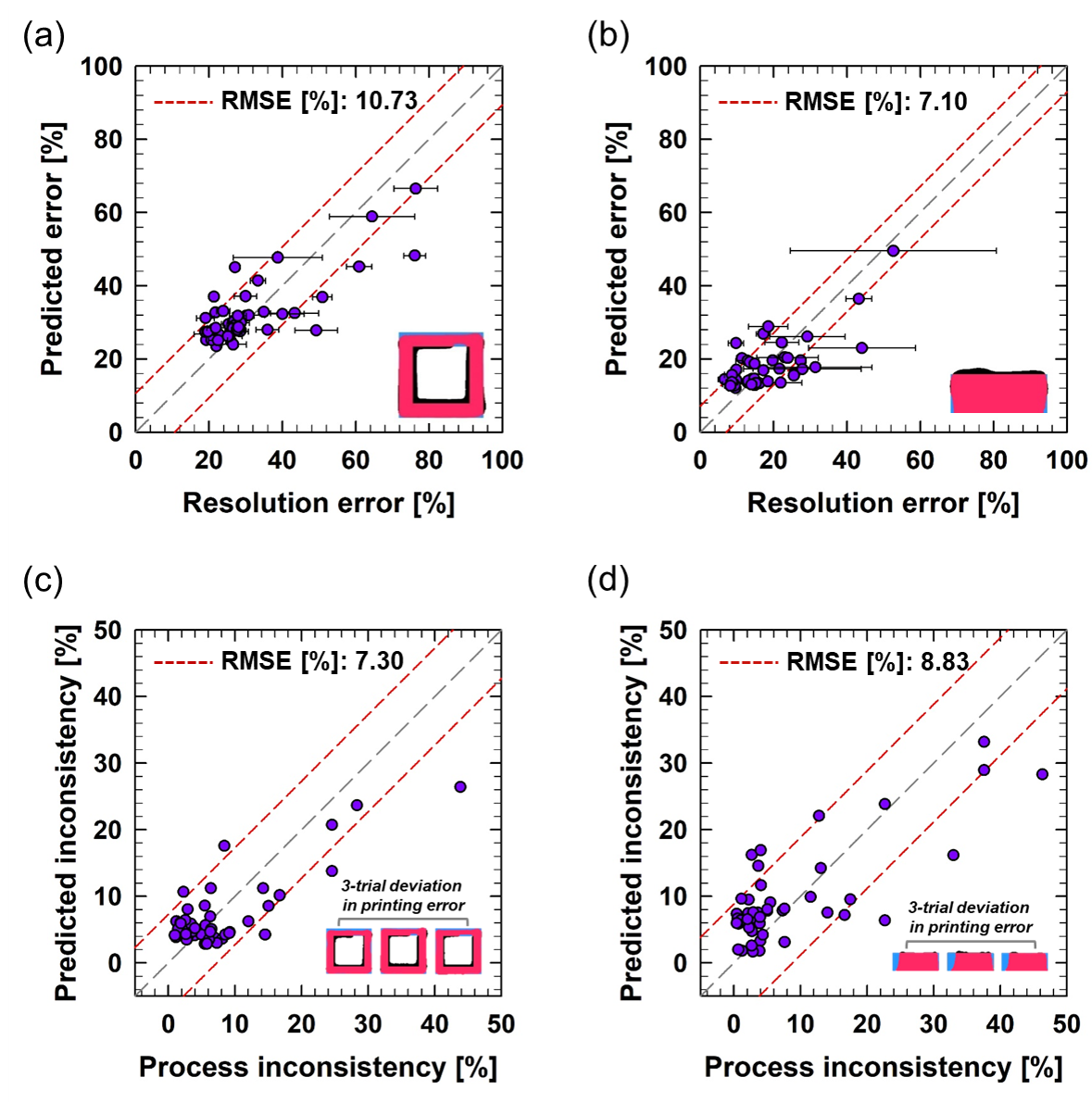


Figure S2. Independent validation of the 3D printability prediction model. Prediction performance of the model evaluated on an independent validation set, based on a 70:30 train-test split. The model was first trained on 70% of the dataset using a fivefold cross-validation approach for model development, and its predictive performance was then assessed on the remaining 30% independent validation set. Prediction performance of the model for (a) horizontal printing error, (b) vertical printing error, (c) printing inconsistency in the horizontal direction, and (d) printing inconsistency in the vertical direction. The dashed red lines represent the RMSE deviation range, while the gray long-dashed lines indicate the ideal prediction line (y = x). Error bars indicate deviations obtained from three repeated measurements.

**Table S1. Detailed composition of AAm-Laponite based Hydrogel inks.**All compositions are given in wt%

| **No.** | **Laponite** | **AAm** | **Irgacure** | **BIS** | **DI Water** |
| --- | --- | --- | --- | --- | --- |
| **1** | 6.641 | 7.882 | 0.830 | 1.660 | 82.987 |
| **2** | 7.894 | 7.776 | 0.819 | 1.638 | 81.873 |
| **3** | 10.714 | 7.538 | 0.794 | 1.588 | 79.367 |
| **4** | 8.705 | 7.707 | 0.812 | 1.624 | 81.152 |
| **5** | 5.469 | 7.981 | 0.841 | 1.681 | 84.029 |
| **6** | 7.254 | 7.830 | 0.825 | 1.649 | 82.442 |
| **7** | 6.040 | 7.932 | 0.836 | 1.671 | 83.521 |
| **8** | 6.614 | 7.849 | 1.240 | 1.653 | 82.644 |
| **9** | 6.669 | 7.914 | 0.417 | 1.667 | 83.332 |
| **10** | 5.696 | 6.760 | 0.712 | 1.424 | 85.408 |
| **11** | 7.963 | 9.450 | 0.995 | 1.991 | 79.602 |
| **12** | 6.538 | 9.315 | 0.817 | 1.635 | 81.696 |
| **13** | 6.748 | 6.402 | 0.843 | 1.687 | 84.319 |
| **14** | 6.438 | 10.695 | 0.805 | 1.610 | 80.452 |
| **15** | 6.857 | 4.885 | 0.857 | 1.714 | 85.686 |
| **16** | 6.599 | 7.831 | 0.825 | 2.286 | 82.459 |
| **17** | 6.685 | 7.934 | 0.836 | 1.007 | 83.538 |
| **18** | 6.131 | 7.275 | 0.766 | 1.533 | 84.295 |
| **19** | 7.240 | 8.592 | 0.905 | 1.810 | 81.452 |
| **20** | 5.351 | 7.955 | 1.257 | 1.676 | 83.762 |
| **21** | 7.862 | 7.744 | 1.224 | 1.631 | 81.539 |
| **22** | 8.773 | 7.681 | 1.052 | 1.618 | 80.876 |
| **23** | 5.382 | 8.002 | 0.674 | 1.686 | 84.256 |
| **24** | 7.927 | 7.808 | 0.411 | 1.645 | 82.210 |
| **25** | 9.098 | 7.708 | 0.406 | 1.624 | 81.164 |
| **26** | 7.037 | 10.629 | 0.800 | 1.599 | 79.936 |
| **27** | 7.145 | 9.251 | 0.812 | 1.624 | 81.168 |
| **28** | 7.373 | 6.363 | 0.838 | 1.676 | 83.751 |
| **29** | 8.118 | 4.817 | 0.846 | 1.691 | 84.529 |
| **30** | 7.989 | 6.321 | 0.832 | 1.664 | 83.193 |
| **31** | 7.745 | 9.191 | 0.807 | 1.613 | 80.644 |
| **32** | 7.628 | 10.561 | 0.795 | 1.589 | 79.428 |
| **33** | 9.344 | 4.753 | 0.834 | 1.669 | 83.400 |
| **34** | 9.198 | 6.238 | 0.821 | 1.643 | 82.100 |
| **35** | 8.920 | 9.074 | 0.796 | 1.593 | 79.617 |
| **36** | 6.705 | 7.233 | 0.762 | 1.524 | 83.777 |
| **37** | 6.758 | 6.684 | 0.704 | 1.408 | 84.446 |
| **38** | 7.815 | 11.130 | 0.977 | 1.954 | 78.124 |
| **39** | 8.116 | 7.706 | 1.015 | 2.029 | 81.134 |
| **40** | 7.900 | 9.375 | 0.988 | 2.765 | 78.972 |
| **41** | 8.027 | 9.526 | 1.003 | 1.204 | 80.240 |
| **42** | 6.641 | 7.882 | 1.162 | 1.328 | 82.987 |
| **43** | 6.598 | 7.830 | 1.155 | 1.979 | 82.439 |
| **44** | 7.194 | 9.391 | 0.719 | 1.798 | 80.898 |
| **45** | 8.023 | 8.569 | 1.203 | 2.006 | 80.199 |
| **46** | 7.808 | 11.119 | 1.074 | 1.952 | 78.048 |
| **47** | 7.281 | 7.855 | 0.827 | 1.324 | 82.712 |
| **48** | 7.904 | 7.817 | 0.823 | 1.153 | 82.303 |
| **49** | 8.438 | 7.703 | 0.811 | 1.947 | 81.101 |
| **50** | 8.507 | 7.766 | 0.818 | 1.145 | 81.765 |

**Table S2. Detailed composition of NIPAM-Laponite based Hydrogel inks.**All compositions are given in wt%

| **No** | **Laponite** | **NIPAM** | **pNIPAM** | **Irgacure** | **DI Water** |
| --- | --- | --- | --- | --- | --- |
| **1** | 6.756 | 8.443 | 0.200 | 0.170 | 84.431 |
| **2** | 8.000 | 8.331 | 0.197 | 0.167 | 83.305 |
| **3** | 5.479 | 8.559 | 0.202 | 0.172 | 85.588 |
| **4** | 6.750 | 8.435 | 0.299 | 0.169 | 84.347 |
| **5** | 6.763 | 8.452 | 0.100 | 0.170 | 84.515 |
| **6** | 6.751 | 8.436 | 0.199 | 0.254 | 84.360 |
| **7** | 6.762 | 8.450 | 0.200 | 0.085 | 84.503 |
| **8** | 8.129 | 10.159 | 0.240 | 0.204 | 81.268 |
| **9** | 5.780 | 7.223 | 0.171 | 0.145 | 86.681 |
| **10** | 7.070 | 8.415 | 0.199 | 0.169 | 84.147 |
| **11** | 6.440 | 8.472 | 0.200 | 0.170 | 84.718 |
| **12** | 6.644 | 9.963 | 0.196 | 0.167 | 83.030 |
| **13** | 6.873 | 6.871 | 0.203 | 0.173 | 85.880 |
| **14** | 6.536 | 11.434 | 0.193 | 0.164 | 81.673 |
| **15** | 6.993 | 5.243 | 0.207 | 0.176 | 87.381 |
| **16** | 7.382 | 8.386 | 0.198 | 0.169 | 83.865 |
| **17** | 6.880 | 8.432 | 0.199 | 0.169 | 84.320 |
| **18** | 7.692 | 8.358 | 0.198 | 0.168 | 83.584 |
| **19** | 5.802 | 8.530 | 0.202 | 0.171 | 85.295 |
| **20** | 7.865 | 9.831 | 0.194 | 0.165 | 81.945 |
| **21** | 8.131 | 6.779 | 0.200 | 0.170 | 84.720 |
| **22** | 5.345 | 10.862 | 0.197 | 0.168 | 83.428 |
| **23** | 5.578 | 6.968 | 0.206 | 0.175 | 87.073 |
| **24** | 6.322 | 7.182 | 0.170 | 0.144 | 86.182 |
| **25** | 8.870 | 10.077 | 0.238 | 0.202 | 80.613 |
| **26** | 5.233 | 7.265 | 0.172 | 0.146 | 87.184 |
| **27** | 7.376 | 10.242 | 0.242 | 0.206 | 81.934 |
| **28** | 9.392 | 8.204 | 0.194 | 0.165 | 82.044 |
| **29** | 5.287 | 8.576 | 0.203 | 0.172 | 85.762 |
| **30** | 8.305 | 8.303 | 0.196 | 0.167 | 83.029 |
| **31** | 5.154 | 8.588 | 0.203 | 0.173 | 85.882 |
| **32** | 6.279 | 7.062 | 0.186 | 0.158 | 86.315 |
| **33** | 6.304 | 6.696 | 0.186 | 0.158 | 86.655 |
| **34** | 7.087 | 6.848 | 0.202 | 0.258 | 85.605 |
| **35** | 6.498 | 7.693 | 0.162 | 0.172 | 85.476 |
| **36** | 5.508 | 8.173 | 0.203 | 0.086 | 86.030 |
| **37** | 5.866 | 5.864 | 0.156 | 0.147 | 87.966 |
| **38** | 8.070 | 7.563 | 0.159 | 0.169 | 84.039 |
| **39** | 5.283 | 9.430 | 0.223 | 0.190 | 84.874 |
| **40** | 5.372 | 7.672 | 0.453 | 0.193 | 86.310 |
| **41** | 7.929 | 7.340 | 0.174 | 0.147 | 84.410 |
| **42** | 7.417 | 8.341 | 0.548 | 0.279 | 83.415 |
| **43** | 7.955 | 7.646 | 0.181 | 0.108 | 84.110 |
| **44** | 6.049 | 7.199 | 0.170 | 0.188 | 86.394 |
| **45** | 8.274 | 8.685 | 0.156 | 0.166 | 82.719 |
| **46** | 5.780 | 8.923 | 0.181 | 0.137 | 84.979 |
| **47** | 6.114 | 6.598 | 0.263 | 0.209 | 86.816 |
| **48** | 5.893 | 7.773 | 0.251 | 0.164 | 85.919 |
| **49** | 7.612 | 8.370 | 0.162 | 0.153 | 83.703 |
| **50** | 8.245 | 7.925 | 0.375 | 0.239 | 83.216 |

**Table S3. Detailed composition of NIPAM-Carbomer based Hydrogel inks.**All compositions are given in wt%

| **No** | **Carbomer** | **NIPAM** | **a-keto** | **BIS** | **NaOH** | **Water** |
| --- | --- | --- | --- | --- | --- | --- |
| **1** | 0.928 | 14.164 | 0.010 | 0.004 | 0.139 | 84.754 |
| **2** | 0.592 | 17.794 | 0.028 | 0.006 | 0.193 | 81.387 |
| **3** | 1.117 | 15.456 | 0.045 | 0.009 | 0.468 | 82.905 |
| **4** | 0.851 | 17.902 | 0.020 | 0.004 | 0.139 | 81.084 |
| **5** | 0.974 | 17.547 | 0.027 | 0.006 | 0.475 | 80.970 |
| **6** | 1.178 | 17.689 | 0.028 | 0.006 | 0.192 | 80.908 |
| **7** | 0.846 | 17.779 | 0.030 | 0.004 | 0.138 | 81.204 |
| **8** | 1.597 | 17.268 | 0.045 | 0.009 | 0.312 | 80.769 |
| **9** | 0.985 | 17.753 | 0.030 | 0.004 | 0.137 | 81.090 |
| **10** | 0.919 | 11.983 | 0.020 | 0.004 | 0.207 | 86.866 |
| **11** | 0.425 | 17.854 | 0.030 | 0.004 | 0.138 | 81.549 |
| **12** | 2.188 | 16.897 | 0.066 | 0.009 | 0.305 | 80.535 |
| **13** | 2.073 | 15.441 | 0.045 | 0.005 | 0.311 | 82.126 |
| **14** | 1.120 | 13.600 | 0.045 | 0.014 | 0.312 | 84.908 |
| **15** | 1.581 | 11.479 | 0.067 | 0.009 | 0.154 | 86.710 |
| **16** | 0.980 | 17.665 | 0.028 | 0.006 | 0.287 | 81.034 |
| **17** | 1.910 | 17.213 | 0.045 | 0.009 | 0.311 | 80.511 |
| **18** | 0.788 | 17.759 | 0.028 | 0.006 | 0.192 | 81.226 |
| **19** | 0.983 | 17.724 | 0.028 | 0.006 | 0.192 | 81.067 |
| **20** | 0.923 | 16.045 | 0.020 | 0.002 | 0.139 | 82.872 |
| **21** | 0.565 | 17.829 | 0.030 | 0.004 | 0.138 | 81.434 |
| **22** | 1.262 | 17.057 | 0.067 | 0.009 | 0.308 | 81.297 |
| **23** | 0.987 | 17.784 | 0.028 | 0.006 | 0.096 | 81.099 |
| **24** | 0.710 | 17.927 | 0.020 | 0.004 | 0.139 | 81.199 |
| **25** | 2.060 | 13.471 | 0.045 | 0.014 | 0.309 | 84.101 |
| **26** | 1.881 | 16.950 | 0.066 | 0.009 | 0.306 | 80.788 |
| **27** | 0.964 | 17.379 | 0.045 | 0.010 | 0.314 | 81.288 |
| **28** | 0.497 | 12.034 | 0.020 | 0.004 | 0.208 | 87.237 |
| **29** | 0.956 | 17.228 | 0.067 | 0.006 | 0.187 | 81.557 |
| **30** | 2.055 | 15.310 | 0.044 | 0.009 | 0.463 | 82.118 |
| **31** | 0.982 | 17.690 | 0.028 | 0.009 | 0.192 | 81.100 |
| **32** | 2.077 | 11.603 | 0.045 | 0.009 | 0.156 | 86.109 |
| **33** | 1.127 | 13.686 | 0.045 | 0.005 | 0.314 | 84.823 |
| **34** | 0.920 | 14.046 | 0.020 | 0.006 | 0.138 | 84.869 |
| **35** | 2.221 | 17.159 | 0.045 | 0.009 | 0.310 | 80.256 |
| **36** | 1.282 | 17.324 | 0.045 | 0.010 | 0.313 | 81.027 |
| **37** | 0.949 | 17.111 | 0.067 | 0.009 | 0.309 | 81.555 |
| **38** | 1.371 | 17.655 | 0.028 | 0.006 | 0.191 | 80.749 |
| **39** | 0.992 | 17.876 | 0.020 | 0.004 | 0.138 | 80.969 |
| **40** | 0.771 | 12.133 | 0.011 | 0.005 | 0.075 | 87.005 |
| **41** | 0.569 | 17.953 | 0.020 | 0.004 | 0.139 | 81.315 |
| **42** | 0.980 | 17.656 | 0.023 | 0.010 | 0.319 | 81.012 |
| **43** | 2.256 | 17.429 | 0.023 | 0.010 | 0.315 | 79.968 |
| **44** | 0.974 | 17.556 | 0.041 | 0.006 | 0.190 | 81.233 |
| **45** | 0.499 | 16.113 | 0.020 | 0.002 | 0.139 | 83.226 |
| **46** | 0.431 | 18.104 | 0.010 | 0.004 | 0.140 | 81.311 |
| **47** | 0.978 | 17.623 | 0.028 | 0.014 | 0.191 | 81.166 |
| **48** | 0.428 | 17.978 | 0.020 | 0.004 | 0.139 | 81.431 |
| **49** | 1.170 | 17.572 | 0.027 | 0.006 | 0.381 | 80.844 |
| **50** | 0.498 | 14.106 | 0.020 | 0.006 | 0.139 | 85.231 |
